# Flagellar motility and the mucus environment influence aggregation-mediated antibiotic tolerance of *Pseudomonas aeruginosa* in chronic lung infection

**DOI:** 10.1128/mbio.00831-25

**Published:** 2025-05-15

**Authors:** Matthew G. Higgs, Matthew A. Greenwald, Cristian Roca, Jade K. Macdonald, Ashelyn E. Sidders, Brian P. Conlon, Matthew C. Wolfgang

**Affiliations:** 1 Department of Microbiology and Immunology, University of North Carolina at Chapel Hill 2331, Chapel Hill, North Carolina, USA; 2 Marsico Lung Institute, University of North Carolina at Chapel Hill 2331, Chapel Hill, North Carolina, USA; University of Washington, Seattle, Washington, USA

**Keywords:** *Pseudomonas aeruginosa*, flagellar motility, aggregation, antibiotic efficacy, muco-obstructive airway diseases

## Abstract

*Pseudomonas aeruginosa* routinely causes chronic lung infection in individuals with muco-obstructive airway diseases (MADs). In MADs, *P. aeruginosa* forms antibiotic-tolerant biofilm-like aggregates within hyperconcentrated airway mucus. While the contribution of mucin hyper-concentration to antibiotic tolerance and bacterial aggregation has been described, less is known about the bacterial factors involved. We previously found that *P. aeruginosa* populations isolated from people with MADs exhibited significant variability in antibiotic tolerance. This variability is not explained by antibiotic resistance or the mucus environment, suggesting bacterial-driven mechanisms play a crucial role in treatment outcomes. Here, we investigated the contribution of flagellar motility to aggregate formation and tolerance by manipulating motility behaviors. Similar to prior studies, we found that loss of flagellar motility resulted in increased aggregation and tolerance to various antibiotics. We identified novel differential roles of the MotAB and MotCD stators, which power flagellar rotation, in antimicrobial tolerance and aggregate formation. In addition, we found that control of *fliC* expression was important for aggregate formation and antibiotic tolerance. Constitutive expression of *fliC* allowed *P. aeruginosa* to overcome entropic forces of mucin, antagonizing aggregate formation and increasing antibiotic efficacy. Lastly, we demonstrate that neutrophil elastase, an abundant antimicrobial protease in chronic lung infection, promotes antibiotic treatment failure by impairing flagellar motility leading to antibiotic-tolerant aggregate formation. These results underscore the crucial role of flagellar motility in aggregate formation and antibiotic tolerance, enhancing our understanding of how *P. aeruginosa* adapts to the MADs lung environment*.*

**IMPORTANCE:**

Antibiotic treatment failure of *Pseudomonas aeruginosa* infection is a key driver of mortality in muco-obstructive airway diseases (MADs). The bacterial mechanisms that contribute to antibiotic tolerance in MADS infection are poorly understood. We investigated the impact of swimming motility behaviors on *P. aeruginosa* antibiotic tolerance in the context of the diseased mucus environment. Loss of flagellar motility, a common adaptation in chronic lung infection, drives antibiotic tolerance by promoting aggregate formation under physiologically relevant mucin concentrations. We uncovered novel roles of the flagellar stators in motility and mucus aggregate formation. Furthermore, neutrophil elastase, an abundant host-derived antimicrobial protease, promotes antibiotic tolerance and aggregation by impairing flagellar motility. These results further our understanding of the formation of antibiotic-tolerant aggregates within the MADs airway, revealing potential new targets to improve antibiotic treatment of chronic *P. aeruginosa* airway infection.

## INTRODUCTION

*Pseudomonas aeruginosa* is a Gram-negative bacterium capable of causing a wide range of opportunistic infections (1). Collectively, muco-obstructive airway diseases (MADs) represent the third leading cause of death worldwide, primarily owing to chronic obstructive pulmonary disease (COPD) and the increasing prevalence of bronchiectasis (2). Individuals with MADs such as cystic fibrosis (CF), COPD, and non-CF bronchiectasis (NCFB) frequently suffer from recurrent and chronic *P. aeruginosa* lung infections (3). Antibiotic treatment failure remains a major cause of decreased quality of life and early mortality in individuals with MADs.

While high-dose inhaled antibiotic therapies show increased efficacy and reduced toxicity compared to traditional delivery methods (e.g., oral and intravenous), chronic *P. aeruginosa* infections remain recalcitrant to antibiotic therapy and develop clinical resistance at high frequency (4, 5). While genetically encoded antibiotic resistance remains a global health threat, the failure of antibiotics to eradicate chronic *P. aeruginosa* lung infections in the absence of resistance is primarily due to antibiotic tolerance and persistence (6, 7). Antibiotic tolerance is a physiological state in which bacteria exhibit reduced susceptibility to antibiotic-mediated killing without an accompanying shift in minimum inhibitory concentration (MIC). By contrast, persistence denotes a distinct subpopulation of cells capable of enduring antibiotic exposure for prolonged durations (8). There are many factors that have been shown to contribute to antibiotic tolerance, ranging from environmental and growth conditions to bacterial-encoded factors. For example, MADs are characterized by the accumulation of dehydrated mucus within small airways. We and others have previously shown that airway mucin and DNA size and concentration, which vary greatly between individuals, influence *P. aeruginosa* tolerance to tobramycin (9, 10). Understanding the mechanisms that contribute to antibiotic tolerance and persistence is paramount to the development of more effective treatment strategies.

Stagnant mucus provides a unique, nutrient-rich environment for *P. aeruginosa* colonization that promotes the formation of bacterial community structures known as aggregates (11–13). These aggregates exhibit biofilm-like properties, including enhanced tolerance and resistance to antibiotics, and are associated with antibiotic treatment failure (14). The requirements for aggregation and events that lead to the formation of aggregates are poorly understood. Evidence suggests that the mucus polymer environment is a strong driver of aggregation (15, 16). However, there is also strong evidence that aggregation is not solely dictated by the environment, but rather it is likely a combination of bacterial-driven factors and the diseased mucus environment. It was shown that alterations in *P. aeruginosa* LPS O-antigen led to differences in aggregate assembly (17) and provided evidence that bacterial factors, not just the mucin environment, can also dictate aggregation phenotypes. Despite these advances, there is still a lack of understanding of the bacterial-encoded factors that drive aggregation and how these factors combine with the mucus environment to shape bacterial community structure to drive antibiotic treatment failure.

We previously showed that when using a single mucin concentration *in vitro* (2% wt/vol mucin), *in vivo* evolved *P. aeruginosa* populations recovered from MADs sputum displayed significant variability in antibiotic treatment outcomes that were not explained by antibiotic resistance (9). This strongly suggests that the evolution of bacterial-encoded factors significantly contributes to antibiotic tolerance. Clonal *P. aeruginosa* populations undergo significant genotypic and phenotypic diversification during chronic adaptation within the airway. The chronic infection environment is highly stressful and promotes the accumulation of mutations that drive diversification; chronic isolates are often found to contain mutations in DNA repair mechanisms, making them hypermutable (18, 19). While chronic infection results in a remarkable array of diversity, there are some common evolutionary traits that are selected for in the MADs airway environment. Isolates from chronic infection often exhibit a loss of acute virulence factors and increased antibiotic tolerance, persistence, and antibiotic resistance (20, 21). In addition, the development of mucoid phenotypes and loss of Las quorum sensing are traits commonly found in MADs clinical isolates (22). One of the most frequent adaptations is loss or alteration of flagella-mediated swimming motility (22–24).

The flagellum is a complex macromolecular structure made of dozens of parts. Flagellin, the major subunit of the flagellum fiber, is encoded by *fliC*. Flagellin is a potent pro-inflammatory TLR5 agonist, and swimming motility has been shown to stimulate the formation of neutrophil extracellular traps, which entangle and destroy pathogens (25, 26). It has been suggested that the loss of flagella is an adaptation that may allow for host immune evasion (27, 28). However, the loss of flagellin was found to increase virulence in a mouse model of infection (29). FliC is the final flagellar component to be produced once all other structural components have been assembled (30). As a result, loss of upstream structural components such as the hook filament junction proteins (FlgK, FlgL) or the hook itself (FlgE) will result in the loss of *fliC* expression and ablation of flagellum synthesis.

Several studies have investigated the relationship between loss of flagella, antibiotic susceptibility, and aggregate formation of *P. aeruginosa* under non-physiological laboratory conditions (31–33). For instance, Demirdjian et al. showed that, similar to a wild-type (WT) strain, a *fliC* mutant of PAO1 formed aggregates in LB medium, despite the lack of appreciable surface attachment. The aggregates formed by both their WT and Δ*fliC* strains were similarly more tolerant to antibiotics than planktonic cultures (33). In another study, a mutant lacking the flagellar hook protein (FlgE) displayed altered biofilm structure and a reduction in gentamicin penetration of the biofilm (32). Similarly, a *flgK* mutant was more tolerant to the clinically relevant antibiotic tobramycin and more readily formed aggregates in an agar gel (31). It was also shown that in a mixed culture, flagellar mutants were more fit in the presence of antibiotics (31). Despite these observations, it is unknown if the MADs diseased mucus environment imparts unique properties to flagellar motility, the development of antibiotic tolerance, and the formation of multicellular aggregates.

Flagellar rotation in *P. aeruginosa* is powered by two stator complexes, MotAB and MotCD. Historically, these stators were described to have overlapping function; that is, *P. aeruginosa* is still able to swim if one is absent (34, 35). However, recent studies have begun to reveal functional differences between MotAB and MotCD. On average, MotAB stator units are more abundant within a cell and, as a result, provide higher total torque than MotCD (36, 37). MotCD, however, was found to provide a more stable swimming speed and support swimming in higher viscosity environments, such as agar, polyethylene glycol (PEG), and ficoll (34, 35, 38). In WT cells, the function of MotAB and MotCD is suggested to be additive, as Wu et al. found that the combined total torque generation of individual stator mutants was similar to WT (39). However, in cells lacking MotAB, it was found that MotCD stators are more active (36), and this potentially suggests a mechanism for compensation in the event that one of the stator units becomes defective. While the specific function of the dual stators in *P. aeruginosa* remains to be fully determined, it appears they provide a mechanism to adapt motility behaviors in changing or heterogeneous environments. In addition, the role of the flagellar stators in aggregate formation and antibiotic susceptibility in mucus is unexplored.

Here, we investigated the role of flagellar motility in antibiotic tolerance and aggregate formation in the context of a MADs-like mucus environment. Similar to previous studies, we found that mutants of the flagellar machinery are significantly more tolerant to antibiotic challenge, and increased antibiotic tolerance was associated with aggregate formation. Expanding on this observation, we found that the proportion of motile cells within a population prior to the formation of aggregates likely shapes their aggregate-forming ability. We uncovered novel roles of the MotAB and MotCD stators in flagellar motility in the context of high mucin concentrations. The loss of MotAB resulted in increased motility in mucin, decreased aggregate formation, and decreased tolerance to tobramycin. Whereas deletion of *motCD* resulted in increased aggregation and antibiotic tolerance and decreased motility. We also found that regulation of flagellin (*fliC*) expression is important for aggregate formation, as constitutive expression of *fliC* resulted in poor aggregate formation and hypersusceptibility to tobramycin. Our results significantly increase our understanding of the requirements of aggregate formation and further describe the contribution of flagellar motility to antibiotic tolerance in the context of the MADs airway environment, which has implications for naturally occurring bacterial populations that have highly heterogeneous motility phenotypes.

## RESULTS

### Aggregation increases in a mucin concentration-dependent manner and is associated with antibiotic tolerance

Our previous work found that antibiotic tolerance increases as a function of mucin concentration (9). It has also been shown that charged polymers, including mucins, drive the aggregation of *P. aeruginosa* (15, 16). As such, we investigated the relationship between aggregation, mucin concentration, and antibiotic tolerance in synthetic MADs mucus. To assess aggregation and antibiotic tolerance, we used the WT laboratory strain mPAO1 grown in synthetic CF mucus media 2 (SCFM2), a medium that mimics the MADs lung environment (40, 41), and modulated the mucin content. To assess tolerance, bacteria were grown for 8 hours to the late exponential growth phase, followed by treatment with high-dose tobramycin (300 µg/mL) for an additional 24 hours as previously described (9). The tobramycin concentration used represents the average Cmax achieved in sputum during inhaled tobramycin therapy (42, 43). Aggregation was assessed by confocal microscopy using bacteria that chromosomally expressed the fluorescent protein dsRed-Express2 (44). To capture aggregation within the luminal media rather than surface-attached bacteria, confocal images were taken at least 10 µm above the bottom of the growth chamber surface. Aggregates were quantified using Imaris image analysis software (Oxford Instruments) and the surfaces function; surfaces greater than 5 µm^3^ were considered as aggregates (17).

**Fig 1:**
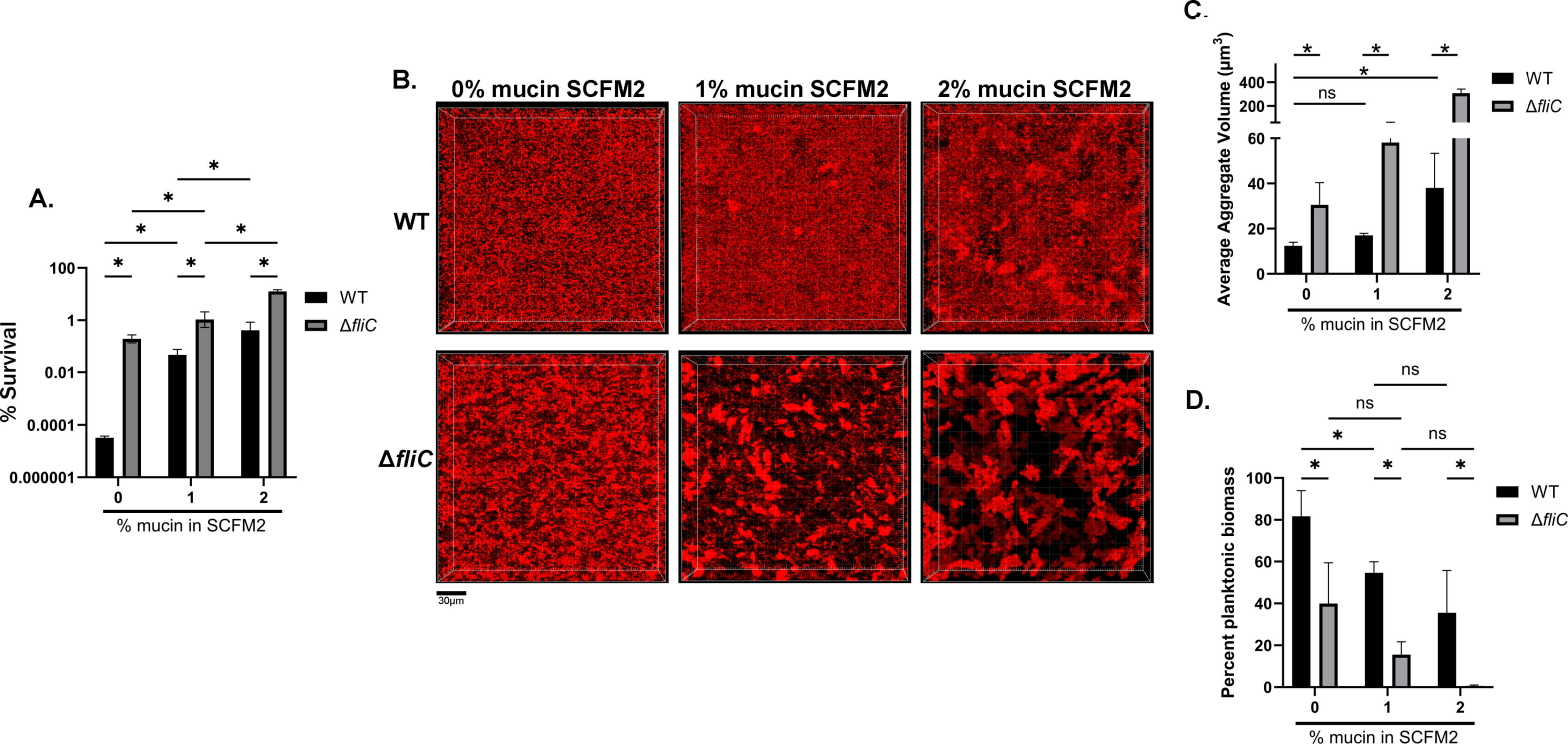
Aggregation is strongly associated with tobramycin tolerance in a mucin concentration-dependent manner. (**A**) WT mPAO1 or Δ*fliC* was grown in SCFM2 with the indicated mucin concentration for 8 hours, then treated with tobramycin (300 µg/mL) for 24 hours. Percent survival is plotted as mean ± SD. (**B**) WT PAO1 or Δ*fliC* chromosomally expressing DsRed-Express2 fluorescent protein was grown in SCFM2 at various mucin concentrations for 6 hours prior to imaging via 3D confocal microscopy. Scale bar is 30 µm. (**C**) Quantification of aggregates from panel B using Imaris. Aggregates were classified as surfaces > 5 µm^3^. Data represent the average median aggregate volume ± SD from at least three independent images. (**D**) Proportion of planktonic biomass (<5 μm^3^) from Imaris analyzed images. Planktonic biomass plotted as average ± SEM from a minimum of three images. All data are representative of three independent experiments. *P < 0.05*,* as determined by two-way ANOVA with Tukey multiple comparisons test (A, D) or multiple Student’s *t-*test (C).

Consistent with our previous work (9), the survival of WT mPAO1 following tobramycin treatment increased significantly as mucin concentration increased (Fig. 1A). While increasing mucin concentration was associated with a general trend toward increased aggregate size, the trend between 0 and 1% (wt/vol) mucin was not significant; a substantial increase to aggregate size was only observed between 0% and 2% (wt/vol) mucin (Fig. 1B, top panels, and C; Fig. S1). More strikingly, we observed a significant decrease in the proportion of planktonic biomass when 1% mucin (wt/vol) was added to SCFM2 (Fig. 1D), indicating that more bacteria were contained within aggregates. Increasing mucin concentration was also associated with a slight increase in the MIC to tobramycin, as we previously reported (9) (Table S1). These data suggest that the introduction of mucin (0% vs. 1% [wt/vol] mucin) primarily causes a shift from planktonic growth to an aggregated state, whereas higher mucin concentrations (1% to 2% [wt/vol]) increase aggregate size. These data also suggest that tolerance to tobramycin is likely dictated by a combination of aggregate size and the proportion of bacteria contained within an aggregate.

### Loss of flagellar motility increases antibiotic tolerance to tobramycin and is associated with increased aggregation

One of the most common phenotypic adaptations observed in chronic *P. aeruginosa* isolates from MADs is the loss of flagellar motility (23, 24). It was previously shown that flagellar mutants exhibit decreased susceptibility to tobramycin when embedded in an agar polymer gel (31). We investigated whether this trend existed in a disease-relevant hyperconcentrated mucus environment. We utilized a non-polar in-frame deletion mutant of *fliC*, which encodes flagellin, the structural subunits of the flagellum fiber. A *fliC* mutant was significantly more tolerant than WT mPAO1 to tobramycin and exhibited a significant increase in aggregation at all mucin concentrations tested (Fig. 1A through C; Fig. S1). The proportion of planktonic biomass also decreased in Δ*fliC* compared to WT, with virtually no planktonic bacteria remaining in SCFM2 with 2% mucin (wt/vol) (Fig. 1D). These data strongly support that aggregation may account for mucin concentration-driven antibiotic tolerance. To confirm the role of the flagellum in tolerance, we also tested an in-frame *flgE* deletion mutant (Δ*flgE*). Both *flgE* and *fliC* mutants fail to produce flagellin or produce surface flagella (Fig. S2A). Similar to the *fliC* mutant, deletion of *flgE* resulted in a significant increase in tobramycin tolerance in SCFM2 with 2% mucin (wt/vol) (Fig. S2B). Furthermore, complementation of *fliC*, expressed from its native promoter, at an ectopic site on the chromosome (Δ*fliC*::ΦCTX-*fliC*) restored both tolerance to tobramycin and aggregation to a level similar to WT (Fig. S2C through E). Given that SCFM2 with 2% mucin (wt/vol) most closely recapitulated *ex vivo* sputum antibiotic tolerance (9), it was used as the standard condition for assays for the rest of this study, unless otherwise noted.

**Fig 2:**
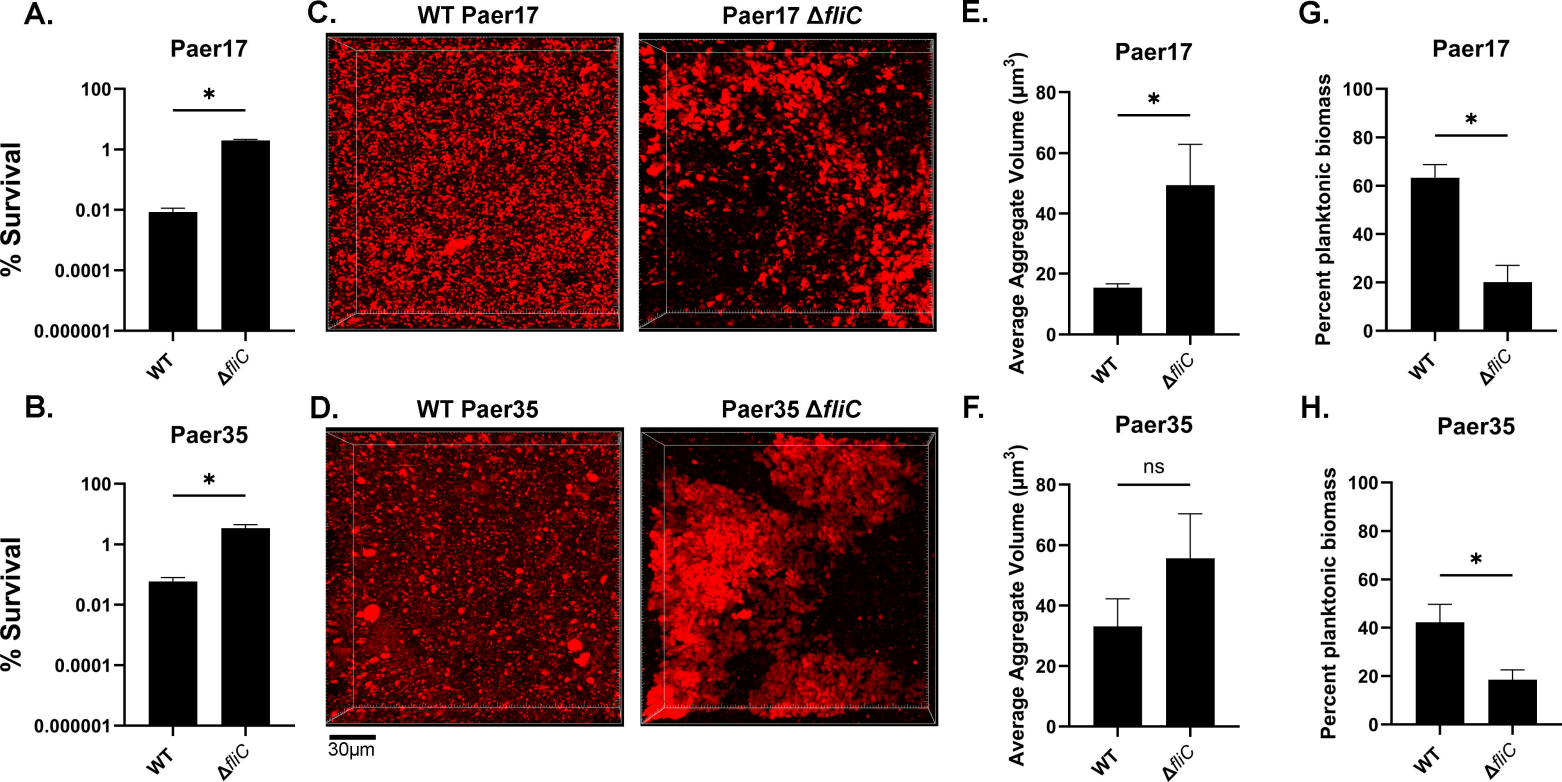
Loss of flagella in *P. aeruginosa* CF clinical isolates promotes tobramycin tolerance and aggregation. Two clinical isolates of *P. aeruginosa* from individuals with cystic fibrosis, along with generated isogenic *fliC* mutants, were assessed for tolerance and aggregation. (**A, B**) Paer17 WT or Δ*fliC* (**A**) or Paer35 WT or Δ*fliC* (**B**) was grown in SCFM2 with 2% mucin (wt/vol) for 8 hours, then treated with tobramycin (300 µg/mL) for 24 hours. Percent survival is plotted as mean ± SD and is representative of three independent experiments. (**C, D**) WT (left panel) or Δ*fliC* (right panel) Paer17 (**C**) or Paer35 (**D**) was grown in SCFM2 with 2% mucin (wt/vol) for 8 hours prior to imaging via 3D confocal microscopy. Scale bar is 30 µm. (**E through H**) Quantification of aggregates (**E, F**) and percent planktonic biomass (**G, H**) from panels C and ), respectively, using Imaris. *P < 0.05 as determined by unpaired Student’s *t-*test. Data are representative of three independent experiments. NS = not significant.

During chronic infection, *P. aeruginosa* displays a remarkable array of diversity, and often results in clinical isolates that are markedly different from laboratory strains. To determine whether the loss of flagella results in increased tolerance and aggregation similar to mPAO1, we utilized two motile CF clinical isolates, designated Paer17 and Paer35, and generated in-frame deletion mutants of *fliC* and assessed tolerance and aggregation phenotypes. Similar to mPAO1, deletion of *fliC* in Paer17 and Paer35 resulted in a significant increase in tolerance to tobramycin (Fig. 2A and B) and increased aggregation (Fig. 2C through H). These results suggest that the loss of flagella leads to an increase in tolerance and aggregation not only in laboratory strains but also in clinical isolates.

**Fig 3:**
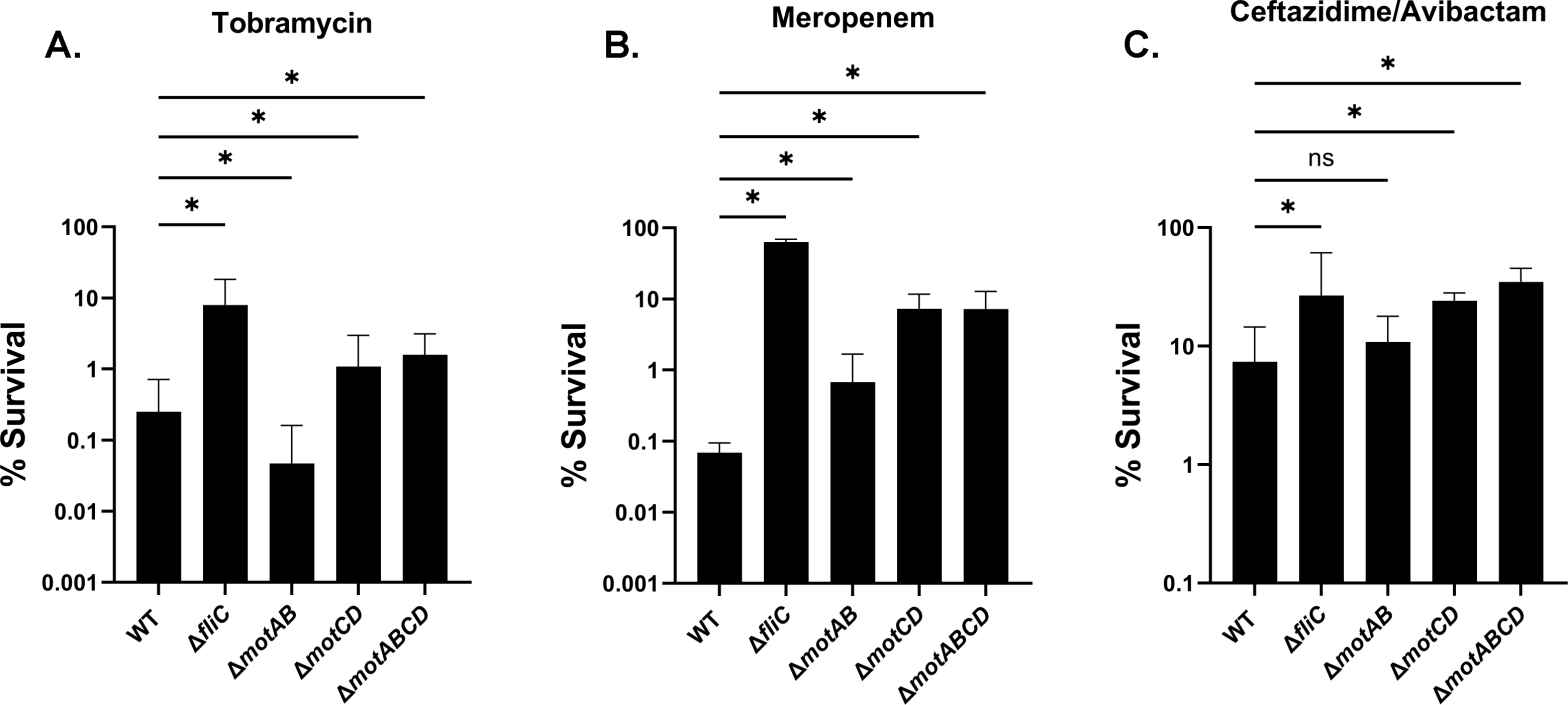
Loss of flagellar motility promotes tolerance to multiple classes of antibiotics. The indicated strains were grown in SCFM2 containing 2% mucin (wt/vol) for 8 hours, then treated with (A) 300 µg/mL tobramycin (aminoglycoside), (B) 2,000 µg/mL meropenem (carbapenem), or (C) 1,000/40 µg/mL ceftazidime/avibactam (third-generation cephalosporin and beta-lactamase inhibitor) for 24 hours. *P < 0.05 as determined by one-way ANOVA with Dunnett’s post hoc test. NS = not significant. Data shown are mean ± SD and are representative of three independent experiments.

We next assessed whether the phenotypes of the *fliC* and *flgE* mutants were related to loss of the flagellum structure or loss of motility. Flagellar rotation is primarily powered by two stator complexes, MotAB and MotCD, with overlapping but distinct function and regulation. As such, we generated non-polar deletion mutants of the *motAB* and *motCD* stators. Deletion of either system alone or both stator complexes simultaneously does not prevent flagellin synthesis or the assembly of flagella on the cell surface (Fig. S2A). Like the non-flagellated mutants, deletion of *motCD* or both stator complexes (Δ*motABCD*) resulted in a significant increase in tolerance to tobramycin (Fig. 3A). However, the *motABCD* mutant did not reach the same level of tolerance as a Δ*fliC* strain, suggesting that loss of the flagellar fiber itself may have an additive effect even in the absence of motility. By contrast, Δ*motAB* exhibited a decrease in tobramycin survival, suggesting a differential role of the stator complexes in antibiotic tolerance.

While tobramycin is one of the most commonly used antibiotics for chronic *P. aeruginosa* lung infection, antibiotics such as meropenem (carbapenem) and ceftazidime/avibactam (cephalosporin/β-lactamase inhibitor) are also frequently used (45–47). Thus, we assessed whether loss of flagellar motility promoted tolerance to other classes of antibiotics. For meropenem and ceftazidime, we similarly used high-dose treatment, approximating the concentrations that could be achieved by inhaled therapies currently in clinical testing (48, 49). Notably, high doses were required to discern differences between strains in the presence of mucin. Like tobramycin, the *motCD* and *motABCD* mutants exhibited an increase in survival compared to WT for both meropenem and ceftazidime/avibactam, similar to Δ*fliC* (Fig. 3B and C). In contrast to the results seen for tobramycin, the *motAB* mutant showed either no difference in tolerance (ceftazidime) or a slight increase (meropenem) compared to WT, demonstrating that the tolerance phenotype of a *motAB* mutant may also be dependent on the antibiotic used, although the same trend for the *motAB* mutant compared to the *motCD* mutant was observed, regardless of the antibiotic used. Despite the observed differences in tolerance of the flagellar mutants to these antibiotics, there was no observed difference in the minimum inhibitory concentration (MIC) of any strain, either in traditional MHB or in SCFM2 (Table 1; Table S1).

**TABLE 1:** MICs for tobramycin, meropenem, and ceftazidime for the PAO1 strain used in this study^*a*^

Strain	Tobramycin	Meropenem	Ceftazidime
WT mPAO1	0.5–1	2–4	2–4
Δ*fliC*	0.5–1	2–4	2–4
Δ*motAB*	0.5–1	2–4	2–4
Δ*motCD*	0.5–1	2–4	2–4
Δ*motABCD*	0.5–1	2–8	2–4
Δ*fliC*::ΦCTX-f*liC*	0.5–1	2–4	2–4
Constitutive *fliC*	0.5–1	2–4	2–4

^*a*^ MICs were measured in Mueller-Hinton broth using standard CLSI criteria. MIC values (μg/mL) are shown. *N* = 3 independent replicates.

The ability of bacteria to survive high concentrations of antibiotics for prolonged periods of time (i.e., persistence) can promote resistance (6, 50). To examine persistence, we assessed the ability of flagellar mutants to withstand prolonged exposures to antibiotics by treating cultures for up to 72 hours (Fig. S3). For all antibiotics tested, both WT and Δ*motAB* had similar susceptibilities, while Δ*fliC* remained tolerant for prolonged periods of time. The *motCD* and *motABCD* mutants exhibited increased tolerance over time compared to both WT and Δ*motAB*, but did not reach the same level of persistence as Δ*fliC*. To determine whether antibiotics remained stable during 72 hours of treatment, we incubated them at 37°C for various times in SCFM2 containing 2% mucin (wt/vol) and then performed MIC assays using WT mPAO1 as a surrogate for antibiotic activity (Table 2). Similar to our previous study (9), we found that the MIC of tobramycin was substantially higher in SCFM2 than in traditional media for assessing MIC (e.g., Mueller-Hinton broth [MHB]) (compare MICs between Tables 1 and 2), likely due to interaction of tobramycin with mucin (51, 52). The activity of meropenem was also substantially reduced in SCFM2 compared to MHB; however, we found that all three antibiotics were generally stable over 72 hours, as there was only a slight change in the MIC of twofold, and likely had little to no impact on survival over 72 hours, though the slight difference may have altered the effective dose of the antibiotics and could result in the slower killing kinetics observed beyond 24 hours.

**TABLE 2:** SCFM2 does not impact antibiotic stability over time^*a*^

Antibiotic	MIC values (μg/mL)		
	No prior incubation in SCFM2	24 hours of incubation in SCFM2	72 hours of incubation in SCFM2
Tobramycin	4–16	4–16	8
Ceftazidime	2–4	2–4	4
Meropenem	16–32	32	64

^*a*^ Antibiotics were added to SCFM2 with 2% mucin (wt/vol) 0, 24, or 72 hours prior to inoculation with WT mPAO1, and a standard MIC assay was performed. *N* = 4 independent replicates.

### Flagellar stators differentially contribute to aggregation

**Fig 4:**
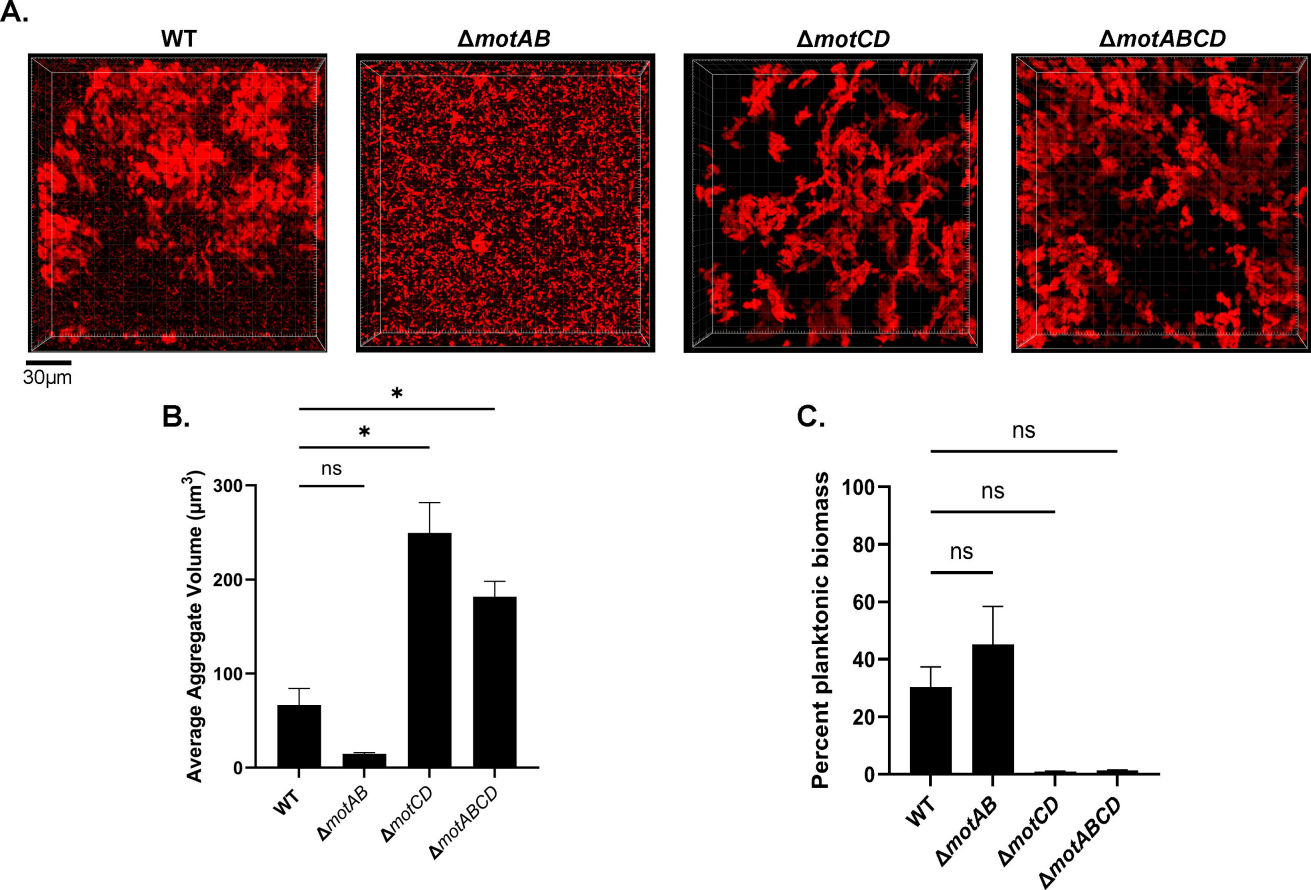
Mutants in flagellar motor rotation significantly impact aggregation. Bacteria were grown in SCFM2 with 2% mucin (wt/vol) for 6 hours before imaging. (**A**) Representative images of motor mutants. Scale bar is 30 µm. (**B**) Imaris quantification of aggregate volumes. (C) Proportion of planktonic biomass of the indicated strains. Data shown are mean ± SD and are representative of three independent experiments. *P < 0.05 as determined by one-way ANOVA with Dunnett’s post hoc test. NS = not significant.

Given that non-flagellated mutants exhibit increased tolerance to several classes of antibiotics and show increased aggregation, we investigated aggregate formation of the stator mutants. We found that the mutants that exhibited increased tobramycin tolerance (Δ*motCD* and Δ*motABCD*) also exhibited a significant increase in aggregate volume compared to WT mPAO1 (Fig. 4A and B). Consistent with the increase in aggregate size, there was a decrease in the proportion of planktonic biomass (Fig. 4C). The Δ*motAB* mutant, which was less tolerant to tobramycin, showed less aggregation than WT and exhibited a higher proportion of planktonic biomass. We also assessed traditional surface-attached biofilm formation by the flagellar mutants in the presence of disease concentrations of mucin (2% wt/vol). Interestingly, we found an inverse association between antibiotic tolerance and surface biofilm formation, where Δ*motAB* exhibited more biofilm formation than WT, and Δ*motCD*, Δ*motABCD*, and Δ*fliC* all exhibited significantly less surface-attached biofilm formation (Fig. S4A). These data support our initial assessment that aggregation is tightly linked to antibiotic tolerance and that the stator complexes have differential roles in promoting aggregate formation in the diseased mucus environment.

### Motile subpopulations are inversely associated with aggregation phenotypes

**Fig 5:**
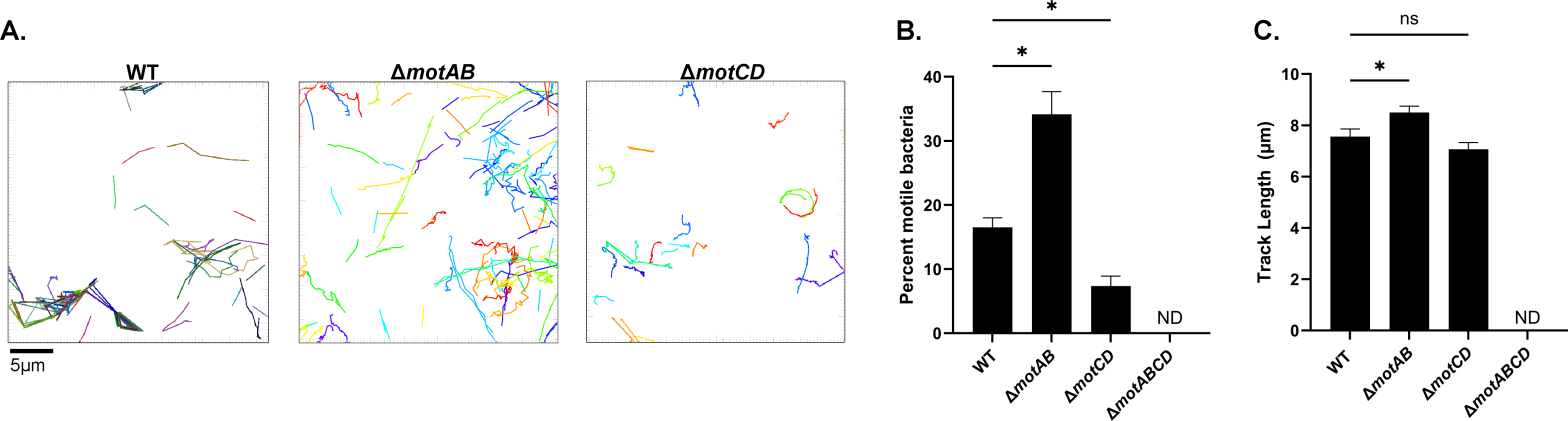
The MotAB and MotCD stators differentially contribute to motility in mucus. Mid-exponential phase bacteria were inoculated into SCFM2 with 2% mucin (wt/vol) and cultured for 1 hour prior to imaging. (**A**) Representative traces of the motile bacteria from each strain, representing one field of view. Scale bar is 5 µm. (**B**) Quantification of the percentage of motile bacteria (>2 µm distance traveled) within the tracked population. (**C**) Average track length for the motile proportion. *P < 0.05 as determined by one-way ANOVA with Dunnett’s post hoc test. NS = not significant. Data shown are mean ± SEM and are representative of two independent experiments and a minimum of 800 tracked cells (**B and C**). ND = no motility detected.

Several studies have investigated the differences between MotAB and MotCD stators in powering flagellar motility. While their general functions are mostly redundant, they do have unique properties under different conditions. MotCD was found to be more important for swarming motility (36). In addition, it was found that while MotAB provides more total rotational torque, MotCD provides better rotational stability (37) and retained functionality under higher load conditions, such as in a viscous environment or near a surface (36, 38). Therefore, we investigated the impact of the stator mutants on motility in our mucin-rich system. As a first step, we assessed swimming motility using a traditional soft-agar-based motility assay. We observed that both Δ*motAB* and Δ*motCD* exhibited a decrease in motility zone diameter compared to WT mPAO1 (Fig. S4B and C). While this agreed with previous studies, it did not explain the decreased tolerance and aggregation result for Δ*motAB* (Fig. 3 and 4). To better discern differences in the stator mutants, we used 2D single-cell motility tracking in our diseased mucus model. We grew bacteria in SCFM2 containing 2% mucin (wt/vol) for 1 hour prior to imaging. This timepoint was chosen to allow sufficient acclimation to the media but also to retain a limited bacterial density to allow for high-resolution imaging of single cells. We observed multiple differences in motility behavior. Most notably, we found a significant difference in the proportion of motile bacteria between WT and the stator mutants. For WT mPAO1, we observed that ~16% of the population was motile at a given time (Fig. 5A and B). The *motAB* mutant exhibited a significant increase in the proportion of motile bacteria compared to WT. Conversely, less than 10% of *motCD* mutant cells were motile at a given time. Despite the significant difference in the motile subpopulation, there were only modest differences in the distance traveled (track length) of the motile bacteria between each strain (Fig. 5A and C). These data suggest that the proportion of motile bacteria within a population may have a direct association with aggregation capabilities.

To better understand the direct impact of mucin on motility, we also conducted single-cell motility tracking with bacteria grown in SCFM2 lacking mucin. In the absence of mucin, we observed a significantly higher proportion of motile bacteria (Fig. S5A), indicating that mucin polymers negatively impact motility. Interestingly, in the absence of mucin, there was no difference in tolerance of Δ*motAB* or Δ*motCD* using high-dose tobramycin (300 µg/mL) (Fig. S5B). However, since these strains were almost completely eradicated using high-dose tobramycin in SCFM2 without mucin, we also assessed their tolerance at a lower concentration of 58 µg/mL, which represents the average serum Cmax for tobramycin (42, 43). Interestingly, both the *motAB* and *motCD* mutants were slightly more tolerant than WT in the absence of mucin at this lower tobramycin concentration (Fig. S5C). Motility tracking showed no difference in the proportion of motile bacteria or track length between WT and stator mutants (Fig. S5D and E). However, both stator mutants exhibited an increase in the proportion of motile bacteria in the absence of mucin compared to SCFM2 with 2% mucin (wt/vol) (Fig. 5; Fig. S5D). Our results indicate that the MotAB and MotCD stators have significantly different roles in motility, aggregation, and tolerance in mucin-rich environments like those encountered during chronic lung infection. In the absence of mucin, the different stator mutants show negligible differences in the behaviors measured.

### FliC regulation is important for tolerance and aggregation

**Fig 6:**
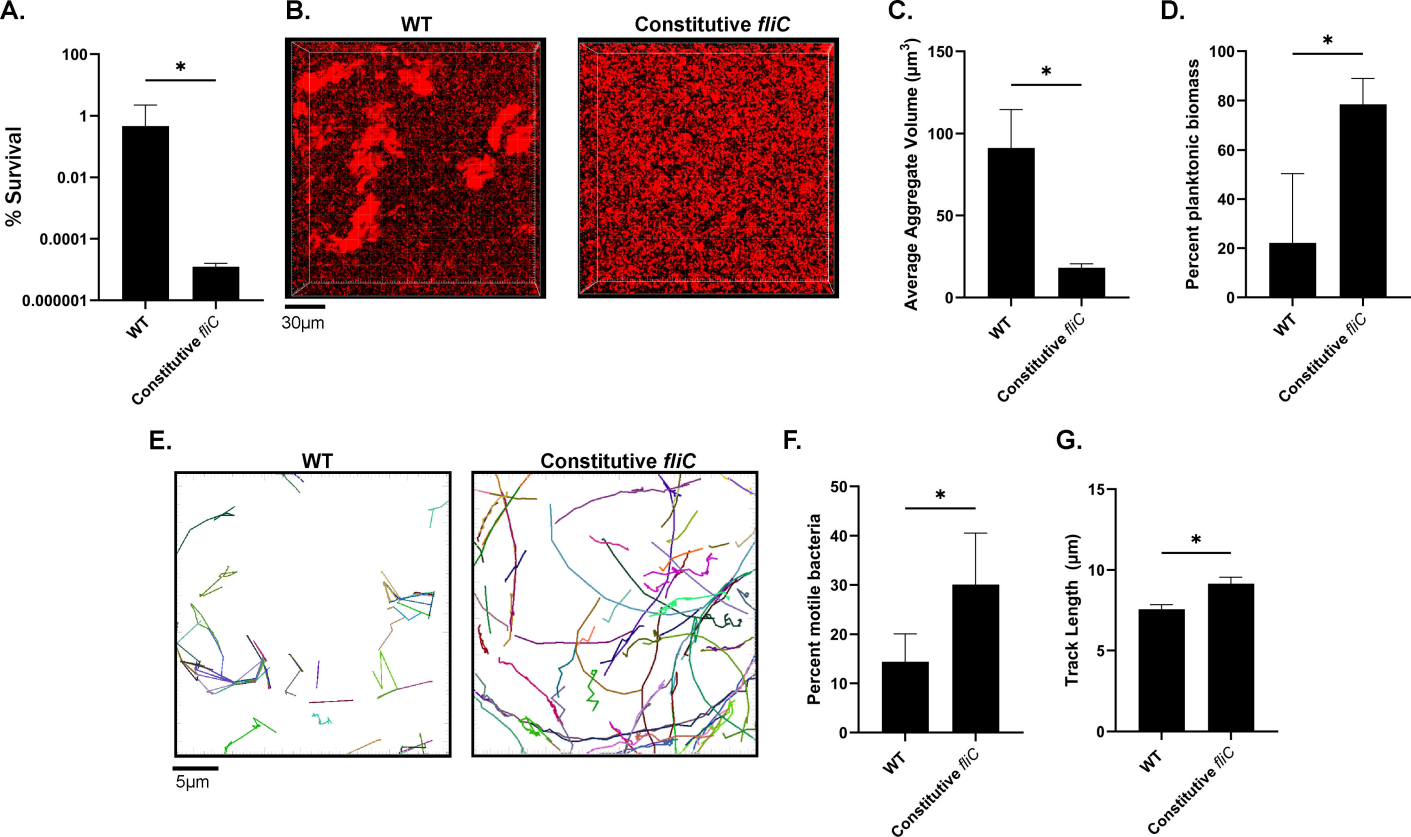
Constitutive *fliC* expression antagonizes tobramycin tolerance and aggregation. (**A**) Survival of WT or constitutive *fliC* expressing strain to tobramycin. (**B**) Representative images of aggregates after growth in SCFM2 containing 2% mucin (wt/vol) for 6 hours. Scale bar is 30 µm. (**C**) Quantification of aggregates from panel B. (**D**) Proportion of planktonic biomass. (**E**) Representative tracking traces of WT or *fliC* constitutive strain as determined by single-cell motility tracking. Scale bar is 5 µm. (**F**) Quantification of the percentage of motile bacteria within the tracked population. (**G**) Average track length for the motile proportion. *P < 0.05 as determined by Student's *t*-test. Data shown are mean ± SD and are representative of three independent experiments (**A through C**), and a minimum of 800 cells tracked (**E through G**).

Downregulation of flagella is important during the development of mature surface-attached biofilms (30, 53, 54) and thus is potentially also involved in mucus-embedded aggregate formation. We engineered a strain expressing *fliC* under an IPTG-inducible Tac promoter, such that flagellin expression is constitutive in the presence of IPTG, and assessed whether the inability to regulate flagellin expression impacted tolerance and aggregation. The addition of 100 µM IPTG restored WT levels of both surface flagellin production and swimming motility in soft agar (Fig. S6). When compared to WT, the constitutive *fliC* strain (100 µM IPTG) exhibited a marked reduction in tolerance to tobramycin (Fig. 6A) and decreased aggregation (Fig. 6B through D). We reasoned that, similar to Δ*motAB*, a constitutive *fliC* strain may have a much higher proportion of motile cells within the population, which would hinder aggregation and therefore decrease tolerance. Indeed, using single-cell tracking, we observed that the constitutive *fliC* strain exhibited a significantly higher proportion of motile cells compared to WT, as well as an increase in track length (Fig. 6E through G). In the absence of IPTG or at low levels (10 μM), the constitutive *fliC* expression strain showed reduced surface flagella and motility in soft agar compared to WT (Fig. S6). At low IPTG concentrations (0 and 10 μM IPTG), the constitutive *fliC* expression strain showed increased tobramycin tolerance compared to WT (Fig. S6C). These data suggest that the regulation of flagella may play a key role in aggregate formation and antibiotic tolerance.

### Neutrophil elastase drives aggregation and antibiotic tolerance

**Fig 7:**
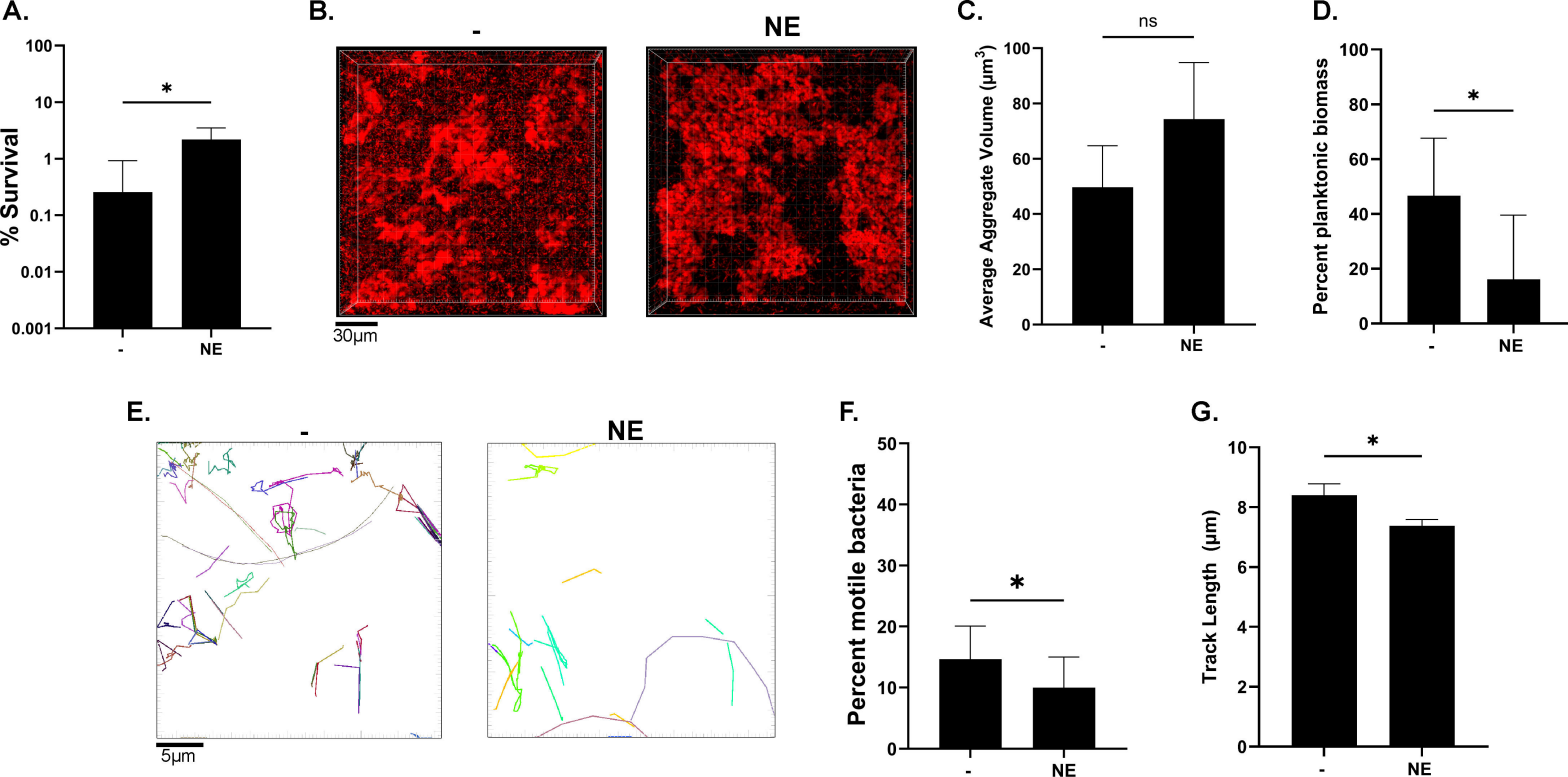
Neutrophil elastase drives aggregation and antibiotic tolerance. (**A**) Bacteria were grown in normal SCFM2 media with 2% mucin (wt/vol) (−) or with neutrophil elastase (NE) (150 µg/mL) for 8 hours and then treated with tobramycin (300 µg/mL) for 24 hours. (**B**) Representative images of aggregates after growth in SCFM2 with 2% mucin (wt/vol) for 6 hours. Scale bar is 30 µm. (**C**) Imaris quantification of aggregate volumes in panel **B**. (**D**) Proportion of planktonic biomass from analysis of panel **B**. (**E**) Representative motility tracking traces of WT or *fliC* constitutive strain as determined by single-cell motility tracking. Scale bar is 5 µm. (**F**) Quantification of the percentage of motile bacteria within the tracked population. (**G**) Average track length for the motile population. *P < 0.05 as determined by Student's unpaired *t*-test. Data shown are mean ± SD and are representative of three independent experiments (**A through C**), and a minimum of 800 cells tracked (**E through G**).

A hallmark of the MADs airway environment is the dominant neutrophil response. As a result, copious amounts of neutrophil effectors, particularly neutrophil elastase (NE), a non-specific serine protease, accumulate in the airway. It has previously been shown that NE is capable of degrading the flagellin of *P. aeruginosa* (55, 56). Consequently, we evaluated whether NE exposure would result in a phenotype similar to that of the flagellar mutants. We added disease-relevant concentrations of NE (150 µg/mL) (57) to SCFM2 containing 2% mucin (wt/vol) and assessed tolerance to tobramycin and aggregation. We observed that exposure to NE led to an increase in tobramycin tolerance and an increase in aggregation (Fig. 7A through C). While the increase in aggregate size did not reach statistical significance (Fig. 7C), there was a significant shift toward a reduced proportion of planktonic biomass in NE-treated samples (Fig. 7D). Exposure to NE also led to a significant decrease in the proportion of motile bacteria and a decrease in track length (Fig. 7E through G). These observations suggest that while aggregate size likely plays a part in antibiotic tolerance, the proportion of bacteria in aggregates of any size (reduction of planktonic bacteria) has an important role in dictating antibiotic tolerance.

To further confirm whether the role of NE in driving tolerance is through its action on flagella, we investigated how loss of the outer membrane porin, OprF, another known target of NE (56), affects tolerance. We found that an *oprF* mutant was highly sensitive to tobramycin (Fig. S7A). In addition, we treated a *fliC* mutant with NE to determine whether there would be any additional increase in tolerance. The *fliC* mutant did not exhibit higher tolerance when treated with NE (Fig. S7B). However, because we may have reached maximal tolerance in our system with the *fliC* mutant, it may have been difficult to see further increases. To address this and further confirm that NE action on flagella is driving tolerance, we lowered the mucin concentration to 1% wt/vol, where we would be better able to see an increase in tolerance. We found that for WT PAO1 treated with NE in SCFM2 with 1% mucin (wt/vol), we still observed an increase in survival; however, the *fliC* mutant did not exhibit any further increase in survival when treated with NE (Fig. S7C). These data suggest that NE exposure drives antibiotic tolerance by impairing flagellar motility, thereby increasing aggregation. Lastly, these data suggest that the host immune response may protect bacteria against antibiotic attack.

## DISCUSSION

In MADs, *P. aeruginosa* infections are challenging to treat due to antibiotic tolerance, which leads to treatment failure. While the mechanisms of antibiotic tolerance in the MADs environment are only partially understood, it is known that environmental factors play a substantial role by promoting aggregate formation. Secor et al. have shown that interaction with charged polymers, such as mucin or eDNA, abundant in the MAD airway environment, drives aggregation by depletion (15, 16). This passive form of aggregation promotes tolerance to antibiotics. More recent studies suggest that environmental factors alone cannot predict antibiotic treatment outcomes. While polymer content within the lungs of individuals with MADs varies greatly and partially explains the diminished efficacy of antibiotics *in vivo*, Greenwald et al*.* have shown that naturally evolved *P. aeruginosa* populations, recovered from people with MADs, exhibit substantial diversity in antibiotic tolerance when tested under identical polymer conditions (9). This suggests that despite the presence of known environmental factors that drive tolerance, bacterial behaviors and bacterial-encoded factors also play an important role. Indeed, Azimi et al*.* have shown that alterations in O-antigen and cell surface hydrophobicity can determine aggregate assembly type, providing direct evidence that bacterial-encoded factors, not just environmental factors, dictate aggregation (17). In this study, we built on existing knowledge of aggregate-driven antibiotic tolerance with an emphasis on investigating motility-associated factors that lead to antibiotic treatment failure.

*P. aeruginosa* primarily resides as aggregates within the MADs lung (11, 12, 58). While the shift to the aggregate lifestyle could be a result of changes in polymer content, bacterial adaptation to chronic infection likely also plays a role. One of the most common evolutionary adaptations observed in *P. aeruginosa* isolates from MADs is loss of flagellar motility (23, 24, 59). Several studies have shown that, despite reduced surface attachment, *P. aeruginosa* strains lacking flagella can still form multicellular aggregates (31, 33, 60). In some systems, flagellar mutants have been shown to exhibit increased tolerance to antibiotics (31, 32). Our data (Fig. 1 to 3; Fig. S1 to S3) support and extend these findings by showing a direct association between flagellar motility, aggregation, and tolerance to antibiotics using a relevant model of MADs disease (SCFM2 with high concentrations of mucin). These results provide strong evidence that bacterial phenotypes directly impact aggregation and antibiotic tolerance. Importantly, aggregation and tolerance of a *fliC* mutant were still mucin concentration-dependent (Fig. 1), highlighting that both the environment and bacterial phenotypes converge to dictate treatment outcomes.

The frequency at which flagellar mutants are observed in chronically adapted MADs clinical isolates is not surprising given our findings that loss of flagellar motility results in a substantial fitness advantage in the presence of antibiotics. Similar findings have been reported for direct competition assays of motile and non-motile clinical isolates from the same subject using agar-based growth conditions that favor bacterial aggregation (31). Notably, we show that deletion of *fliC* in motile clinical isolates recapitulates the increased aggregation and tolerance phenotypes observed in laboratory strains (Fig. 2). However, while the selection of flagellar mutants during chronic infection can likely be attributed to their increased antibiotic tolerance, the exact mechanisms by which flagellar mutants are selected for over time are not well understood. While random mutagenesis likely explains the initial rise of mutants, there is likely a mix of complex factors that lead to the strong selection of isolates defective in flagellar motility. Interaction with host immune factors may accelerate the selection of various phenotypes, including loss of flagellar motility. Neutrophil elastase (NE) is known to specifically degrade flagella (56, 61). We show here that NE-mediated loss of flagellar motility results in increased aggregation and subsequently, tolerance to tobramycin (Fig. 7; Fig. S7A). The assembly and expression of flagella in the face of continuous NE-mediated degradation likely represent a significant energy waste; consequently, flagellar mutants may have a fitness advantage, as has been proposed by others (62). Therefore, the selection of flagellar mutants may be beneficial for *P. aeruginosa* on several fronts: evasion of NE attack and increased tolerance to antibiotics via aggregation. Given the non-productive, and, in many cases, damaging activity of NE in MADs, a novel approach to tackling antibiotic tolerance could be to use inhibitors of NE. NE inhibitors, such as brensocatib and Sivelestat (63–66), present promising novel therapeutic candidates that could prevent further exacerbation of antibiotic tolerance.

Loss of flagella or flagellin expression is likely the most common mechanism for the loss of flagellar motility in the MADs environment. However, the increased propensity for *P. aeruginosa* to aggregate in environments with high polymer content is likely driven in part by the impact of the viscous mucus environment on flagellar motility. In this study, we show a direct association between the proportion of the population that is motile and aggregation as they relate to the presence of mucin (Fig. 1; Fig. S5A, 0 vs. 2% mucin [wt/vol] SCFM2). Our study revealed interesting and unexpected roles for the flagellar stators in polymer-rich conditions that mimic chronic disease. While MotAB and MotCD have been described as having redundant functions in some studies, recent work has begun to highlight their different functions. The MotCD complex has been shown to facilitate motility in higher viscosity environments (67). Therefore, when MotCD was absent (Δ*motCD*), in a viscous solution such as SCFM2 with 2% mucin (wt/vol), it resulted in reduced motility, which then led to increased aggregation and subsequently increased antibiotic tolerance that we observed in our study (Fig. 3 and 4). It has also been shown that cells with only MotCD (i.e., Δ*motAB*) had 10× more active motors than WT or cells with only MotAB (36). This could explain the observed phenomenon that Δ*motAB* had a higher proportion of motile cells, which are less likely to become trapped by mucins (Fig. 5). Recent findings in *P. putida* also highlight different roles of MotAB and MotCD. Similar to our results, it was found that Δ*motCD* mutants formed less motile cells and also formed sessile clusters (aggregates) (67). However, the role of MotAB in *P. putida* was found to be different compared to *P. aeruginosa*. Loss of *motAB* in *P. putida* resulted in strong impairment of motility, whereas in *P. aeruginosa*, the effect is much less robust (67). Here, however, we may have observed a novel behavior of Δ*motAB* in the context of the mucus environment (35–38, 68). Sole reliance on MotCD in a viscous mucin environment appears to allow for better swimming motility, hence why we observed fewer aggregates and more motile cells in this study.

While aggregation-mediated tolerance to antibiotics appears to be largely conserved across multiple classes of antibiotics, including aminoglycosides, carbapenems, and cephalosporins, we observed that a Δ*motAB* mutant, which exhibits less aggregation, exhibited slightly increased survival to meropenem compared to WT (Fig. S3). This indicates that tolerance to meropenem is likely driven by a combination of aggregation and other unknown factors that likely reflect the different mechanism of action of meropenem, a cell wall targeting antibiotic, compared to tobramycin, a protein synthesis inhibitor.

One distinct function that has been described for MotAB is surface sensing. Upon contact with a surface, cyclic-di-GMP (c-di-GMP) rapidly increases in a MotAB-dependent manner (69). It is possible that in our system, “surface sensing” can include non-traditional surfaces, like mucin polymers. C-di-GMP is involved in the regulation of flagellar gene expression via the transcriptional regulator FleQ (70, 71). Therefore, altered c-di-GMP signaling could be influencing flagellar motility phenotypes in a *motAB* mutant. We posit that sensing these non-traditional surfaces may be important for aggregation and that a *motAB* mutant, with defective surface sensing, is less efficient at forming aggregates.

Interestingly, the *motAB* mutant, which exhibited lower tobramycin tolerance, exhibited increased surface-attached biofilm formation in SCFM2 with 2% mucin (wt/vol) (Fig. S4). This contradicts the common assumption that increased biofilm formation would confer more tolerance. However, biofilm development occurs in multiple stages, and different structural characteristics may contribute to varying levels of antibiotic tolerance. While the aggregates we observe are not adhered to a surface, they are likely to share many features of traditional surface-attached biofilms (12, 13, 58, 72). For instance, it has been shown that, similar to surface-attached biofilms, aggregates produce exopolysaccharides (EPS) (11). In addition, despite flagellar mutants exhibiting reduced surface-attached biofilm formation, one study has shown that loss of *fliC* and *motABCD* (though to a lesser extent) increased production of Pel and Psl EPS in a surface-dependent manner (62). Our results showed that a *motABCD* mutant was more tolerant than WT, but not as tolerant to tobramycin as an isogenic *fliC* mutant. Differences in EPS production could explain these results, though testing this was beyond the scope of our study. Previous research by Secor et al*.* and Staudinger et al*.* demonstrated that EPS was not required for aggregate formation or antibiotic tolerance using knockouts of genes responsible for Pel, Psl, and alginate production (15, 31). However, the impact of increased expression of EPS on antibiotic tolerance remains to be explored.

We show here that a combination of environmentally driven and bacterial-encoded factors has significant ramifications on aggregation and antibiotic tolerance phenotypes. However, the dynamics of aggregate formation and dissolution are poorly understood. It is likely that aggregate size, composition, and structure are in various states of flux based on the ability of bacteria to join or leave aggregates. Our results with the constitutive *fliC* strain (Δ*fliC* + pMMB*-fliC*), which was highly sensitive to tobramycin and did not form large aggregates (Fig. 6 ; Fig. S6), suggest that regulation of *fliC* expression may be important for aggregate formation and/or dispersion. Interestingly, our constitutive *fliC* expression strain should retain the ability to downregulate other components of flagellar motility, but the constitutive expression of *fliC* was sufficient to overcome this. It is possible that turning off flagellar motility is a response to becoming trapped in a polymer mesh, but the inability to specifically control *fliC* expression allows bacteria to more readily escape mucin polymer entrapment, which could explain why the constitutive *fliC* strain is significantly more motile within SCFM2 containing 2% mucin (wt/vol) (Fig. 6). Importantly, while many studies highlight loss of motility as a common feature of clinical isolates, several groups have shown that substantial heterogeneity in motility exists, from both a qualitative (motile vs. non-motile) and quantitative perspective (variations in swimming zones in soft agar motility assays) (73, 74). Although motility in soft agar assays does not necessarily reflect motility in mucus, as we show here, these studies suggest that adaptive mutations could enhance motility in mucus, resulting in escape from aggregates and increased spread in the diseased lung environment, albeit, at the expense of antibiotic susceptibility. Regardless, heterogeneity in motility among clinical isolates could help partially explain differences in tolerance at the population level.

While it remains to be seen whether expression of *fliC* is shut off during aggregate formation, Demirdjian et al*.* found that flagellin is still present within aggregates, which indicates that aggregate formation does not immediately lead to shedding of flagella (33). This may be important for aggregate dynamics. Since flagella are retained, reactivation of motility could present an efficient way for bacteria to escape aggregates and spread. Indeed, it has been shown that response to certain mucin glycans can trigger dispersal of surface-attached *P. aeruginosa* (75), but could possibly work in a similar manner for suspended aggregates. Work by the same group suggests that exposure to mucins can also prevent aggregation of free-swimming bacteria and supports the idea that mucins trigger signaling events within *P. aeruginosa* that alter phenotypes (60). However, as mucus polymer content becomes hyperconcentrated with disease progression and severity, entropic forces and bacterial adaptation ultimately result in the shift to aggregate and biofilm lifestyles. Regardless, these results indicate that there are ordered events that lead to aggregate formation, and that flagellar motility likely plays a substantial role in aggregate maintenance in the diseased mucus environment.

Collectively, our results show a strong association between aggregate formation and antibiotic tolerance of *P. aeruginosa* in an *in vitro* mimic of the MADS lung environment. Adaptation to host environmental factors shifts *P. aeruginosa* into a more aggregative state, thereby conferring tolerance to multiple classes of antibiotics. Flagellar motility plays a key role in the formation of aggregates within the diseased mucus environment, and alterations of motility, as exemplified by *motAB* and *motCD* mutants, can skew population aggregation phenotypes. Host immune-derived factors, such as NE, negatively impact motility and help drive antibiotic tolerance. The results of this study shed light on how such a common adaptation is advantageous in the presence of antibiotics and also deepen our understanding of why mutants in flagellar motility are selected for during chronic infection.

## MATERIALS AND METHODS

### Bacterial strains and culture conditions

All bacterial strains and plasmids used in this study are listed in Table S2. Bacteria were swabbed from frozen stocks onto Lysogeny Broth (LB) (Miller) agar and incubated overnight at 37°C. Overnight liquid cultures were inoculated from single colonies and were shaken overnight in LB broth at 250 RPM at 37°C. SCFM2 was prepared as described (9, 40) or purchased from Synthbiome. Where indicated, antibiotics or neutrophil elastase were added. Neutrophil elastase (Innovative Research) was added at 150 µg/mL.

### Generation of mutants, complementation, and constitutive expression plasmids

Primers used in this study are listed in Table S3. Deletion of genes was achieved through SacB-assisted allelic exchange, using the Gateway Cloning (GW) platform (Invitrogen) (76). Fluorescent bacteria were generated via triparental mating of *P. aeruginosa* with *E. coli* containing the pUC18T-mini-Tn7T-Gm-Pc-DsRed-Express2 (44) plasmid and *E. coli* containing the pTNS2 helper plasmid (77). The vector backbone was removed through the flp recombinase (78). Complementation of *fliC* was achieved through amplifying the *fliC* gene and its promoter region (500 bp upstream of the start codon), then using GW to introduce *fliC* and its promoter into a GW-adapted pMini-CTX vector for chromosomal complementation at the neutral ΦCTX site. The vector backbone was removed through the flp recombinase (78). Complementation was confirmed by PCR and swimming motility assays. Constitutive expression of *fliC* was achieved by amplifying the *fliC* gene and introducing it via GW cloning into the pMMB67 vector, which contains the Tac promoter that is inducible by isopropyl β-D-1-thiogalactopyranoside (IPTG)(79). Constitutive expression of *fliC* was achieved through the addition of 100 µM IPTG to culture conditions. IPTG was only added during culture in SCFM2, and not during overnight culture.

### Antibiotic susceptibility assays

Antibiotic susceptibility assays were performed as previously described (9). Briefly, overnight cultures of *P. aeruginosa* were subcultured into fresh LB at a 1:50 dilution and cultured to exponential phase until an OD_600_ of 0.25 was achieved. Exponential phase bacteria were then inoculated 1:100 into SCFM2 for a final inoculum of 1 × 10^6^ CFU/mL. Bacteria were then incubated statically at 37°C for 8 hours. At 8 hours, duplicate wells were collected and serially diluted and plated for CFU at the time of treatment (“At Treat”). Another set of wells was then treated with various antibiotics: tobramycin at 300 µg/mL, ceftazidime/avibactam at 1,000/40 µg/mL, and meropenem at 2,000 µg/mL. All antibiotics were purchased through Sigma Aldrich. Antibiotic challenge went for an additional 24 (standard assay) or up to 72 hours (persistence assays) before bacteria were collected, washed twice in M63 salts, and plated for enumeration. Percent survival was calculated using the following:


$$\%\;Survival=\left(\frac{Post\;treatment\;CFU}{At\;Treat\;CFU}*100\right)$$

### Soft agar swimming motility assays

Bacteria were grown overnight in LB and subcultured for 1 hour at a 1:50 dilution. 2 µL of subculture was inoculated into LB +0.3% agar. Plates were incubated at room temperature for 30 hours before the zone of motility was measured. Imaging of swimming plates was achieved using the iBright FL1500 imager (Applied Biosystems) using the 490–520 (*TRANS*) filter in the visible channel.

### Fluorescence microscopy

Bacteria were prepared the same as for the antibiotic survival assays described above. 1 × 10^6^ CFU/mL was inoculated into 300 µL SCFM2 in 8-well #1.5 coverglass bottom chamber slides (Cell-Vis, Cat# C8-1.5P). After 6 hours of static incubation at 37°C, or 8 hours for the Paer17 and Paer35 clinical isolates, the center of the wells was imaged using a Leica Stellaris5 laser scanning confocal microscope with an environmental box (Okolab) set to 37C for live cell imaging. Dsred-Express2 fluorescence was observed with a white light laser at a laser line of 554 nm at 5% power and detection range of 569–650 nm and gain of 60. Using a 63×, HC PL APO CS2 oil immersion objective with a numerical aperture of 1.4 and a pinhole diameter of 1 Airy Unit (AU), we obtained 180 × 180 × 30 µm 3D Z-stacks at a resolution of 1,024 × 1,024 and scan speed of 600 hz, beginning at least 10 µm above the bottom of the glass. Images were captured using the LAS X software version 4.5 (Leica Microsystems). Raw Leica image files (LIF) were then exported for analysis.

### Motility tracking

Motility tracking was achieved with the same methods as described above for fluorescent microscopy, with some modifications. Bacteria were inoculated into 100 µL SCFM2 at 5 × 10^6^ CFU/mL and grown statically for 1 hour prior to imaging. 2D videos of 30.75 × 30.75 µm were captured at 256 × 256 resolution with a zoom of 6 and bidirectional scanning at a speed of 1,600 Hz (~11.76 frames per second). Pinhole diameter was set to 6.28 AU. Videos were approximately 1 minute long. A minimum of 10 videos were captured per condition/strain, and a minimum of 800 cells were tracked. Video files were then exported for analysis.

### Image analysis

Analysis of aggregates and motility tracking was performed using Imaris 10.0 (Oxford Instruments). LIF files were imported into Imaris. Images were visualized in Imaris as a 3D max projection. For the analysis of aggregates, we created a custom surface creation parameter algorithm. The background subtraction threshold minimum value was set to 15. Surfaces with volumes less than 0.335 µm^3^ or greater than 5,000 µm^3^ were filtered out as artifacts. Surfaces with volumes greater than 5 µm^3^ were considered aggregates. Examples of aggregates are highlighted in Fig. S1A. Planktonic biomass proportion was determined as the ratio of the sum of surface volumes between 0.335 and 5 μm^3^ to the sum of all surfaces calculated.

For motility tracking, we used the spots function to track bacterial motility. The minimum quality threshold was set to 30, and the maximum gap size was set to zero. For motile bacteria, we set a track distance minimum of 2 µm and the track duration between 0.050 and 5.00 seconds. Traces of motile bacteria were highlighted in Imaris and exported.

### Immunoblot of FliC

Visualization of FliC was achieved through western blotting. Strains were inoculated into SCFM2 containing 2% mucin (wt/vol) and grown for 8 hours. For isolation of surface flagella, bacteria were collected and centrifuged at 7,500 × *g* for 3 minutes to pellet the bacteria without shearing the flagella. The supernatant was removed, and pellets were resuspended in 150 µL M63 salts. Using a 1 mL syringe and 25-gauge needle, the suspensions were passed through the needle 20 times. The cells were pelleted at 17,000 × *g* for 2 minutes, and the supernatant containing sheared flagella from the cell surface was collected. Sample protein content was normalized by quantifying protein content of the cell pellets using the bicinchoninic acid (BCA) assay (Thermo Fisher). Samples were adjusted according to cell pellet protein content and run on a pre-cast 10% TGX acrylamide gel (Bio-Rad) with the Precision Plus Protein Dual Color Standards ladder (Bio-Rad) for 1.5 hours at 80 volts. Proteins were then transferred to a PVDF membrane using standard wet transfer at 90 volts for 1 hour at 4C. Following blocking using 5% milk for 1 hour, the membrane was probed with rabbit anti-FliC polyclonal antibody (80) (1:2,000) overnight at 4C, followed by donkey anti-rabbit secondary antibody conjugated to IRDye680 fluorophore (1:20,000; LI-COR Biosciences, Cat# 926-68023) for 2 hours. Bands were visualized on an iBright FL1500 imager (Applied Biosystems) using the X4 (610–660 nm excitation) and M4 (710–730 nm emission) pre-configured filter set for IRDye680.

### Biofilm assay

Biofilm assays were performed using the crystal violet staining method, as previously described (81). Briefly, overnight cultures of bacteria were subcultured at a 1:50 dilution for 1 hour in LB to allow bacteria to enter the exponential phase. 1 × 10^6^ CFU/mL were then inoculated into SCFM2 containing 2% mucin (wt/vol) in tissue-culture treated 96-well plates. Bacteria were incubated statically at 37°C for 24 hours. To measure biofilm, after incubation, plates were washed in water and dried for 2 hours in ambient air before adding 0.1% crystal violet to stain attached bacteria for 15 minutes at room temperature. After staining, plates were washed three times in water and allowed to dry overnight. 95% ethanol was then added to the wells, and the plate was incubated for 15 minutes at room temperature. Well contents were transferred to a clean 96-well plate, and the absorbance at 550 nm was measured using a Tecan Infinite M Plex plate reader. Wells containing sterile media were used as blanks and negative controls.

### MIC assays

MIC assays were conducted in accordance with standards set forth by the Clinical and Laboratory Standards Institute (CLSI) using the broth microdilution method (82). Briefly, 5 × 10^5^ CFU were inoculated into 100 µL of cation-adjusted Mueller-Hinton Broth (MHB) containing varying dilutions of antibiotics for 24 hours. For MIC assays in SCFM2, after 24 hours of growth, 0.015% resazurin was added, then incubated for an additional 24 hours, as described (83).

### Statistical analysis

All experiments were performed in at least biological triplicate, across different days and media preparations. Statistical analysis was achieved via unpaired Student’s two-tailed *t-*test, one-way analysis of variance (ANOVA), or two-way ANOVA as indicated. Differences were considered significantly different with a *P*-value < 0.05. Statistical tests were carried out using GraphPad Prism version 10.2.

## Data Availability

*P. aeruginosa* CF clinical isolate Paer17 and Paer35 genomes are available in the NCBI database using accession numbers CP080281 and CP089064, respectively, or the following URLs: Paer17, https://www.ncbi.nlm.nih.gov/nuccore/CP080281; Paer35, https://www.ncbi.nlm.nih.gov/nuccore/CP089064. Custom Imaris creation parameters used for the analysis of aggregates and motility tracking are publicly available in the Carolina Digital Repository at https://doi.org/10.17615/tvvm-8f83, or searchable under the ID tvvm-8f83.

## SUPPLEMENTAL MATERIAL

Supplemental Figures and Tables mbio.00831-25-s0001.pdf Figures S1-S7 and Tables S1-S3.

ASM does not own the copyrights to Supplemental Material that may be linked to, or accessed through, an article. The authors have granted ASM a non-exclusive, world-wide license to publish the Supplemental Material files. Please contact the corresponding author directly for reuse.
