# Supplementary material for: Flagellar motility and the mucus environment influence aggregation-mediated antibiotic tolerance of Pseudomonas aeruginosa in chronic lung infection
Source: mBio. 2025 May 15;16(6):e00831-25. doi: 10.1128/mbio.00831-25 (PMC12153314; doi:10.1128/mbio.00831-25)
Supplement: Supplemental Figures and Tables — Figures S1-S7 and Tables S1-S3. [file mbio.00831-25-s0001.pdf]

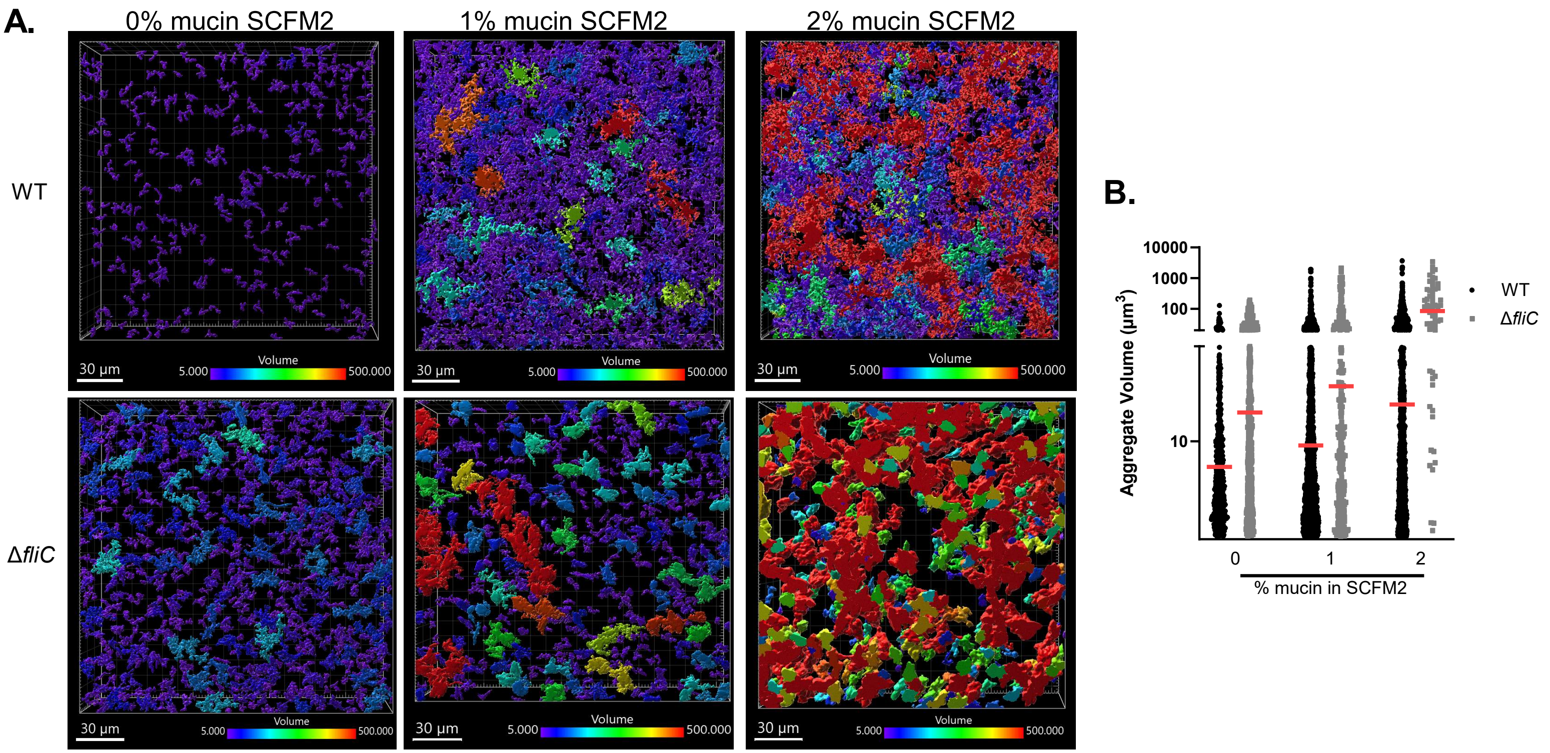

Figure S1. Visualization of Imaris calculated aggregate volumes. A) Representative images from Figure 1 with individual aggregate surfaces highlighted using Imaris. Scale ranges from 5-500µm^3. **B)** Aggregate distribution by size from a single image, plotted on a log scale. Red line indicates median.

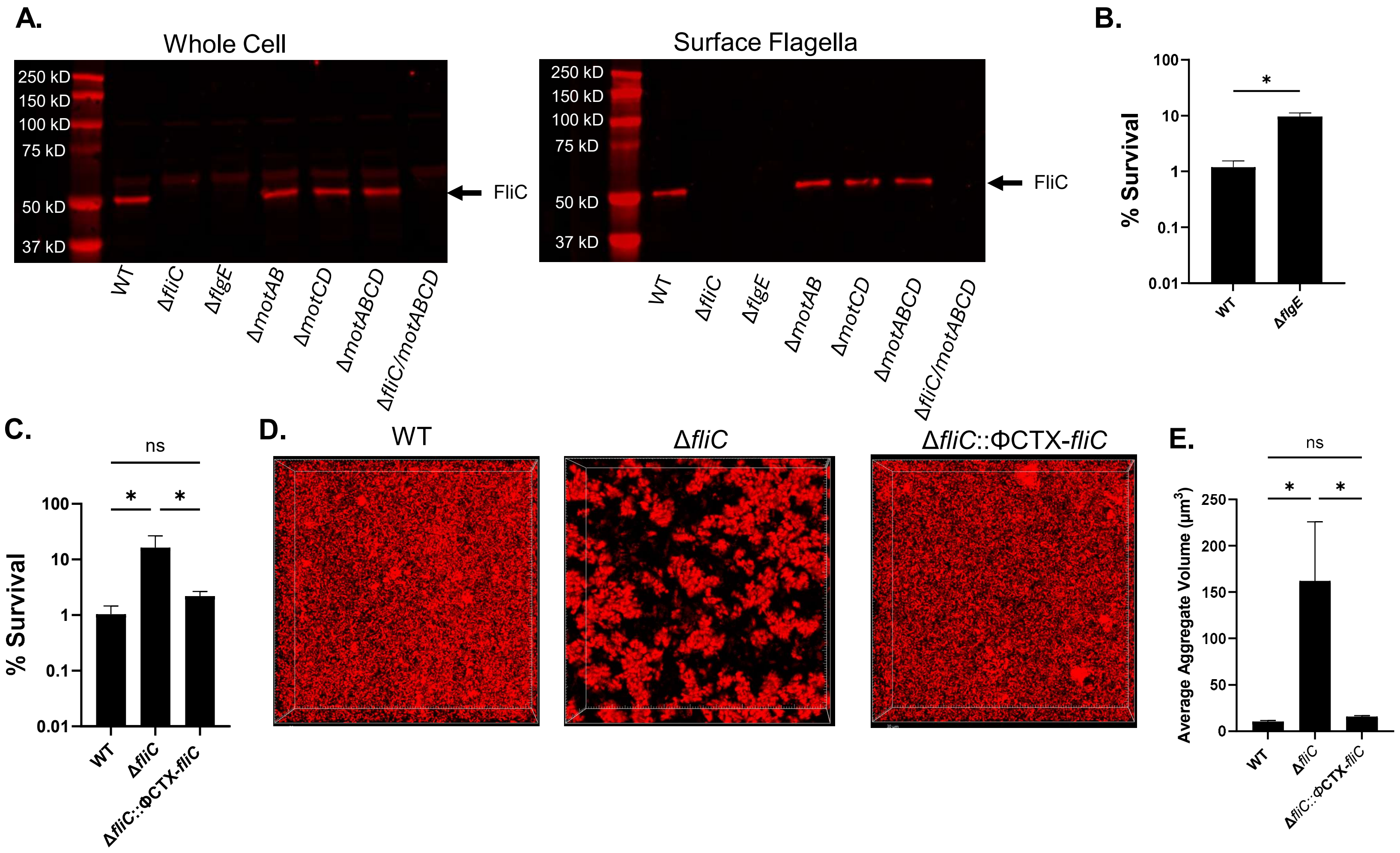

Figure S2. Native *fliC* complementation restores tolerance and motor protein mutants still produce surface flagella. A) Bacteria were grown in SCFM2 containing 2% mucin (w/v) for 8 hours. Whole cell and sheared surface fraction were probed for FliC via western blot **B)** WT mPAO1 and Δ*flgE,* and **C)** WT mPAO1, Δ*fliC*, or *fliC* native complement (Δ*fliC::*ΦCTX-*fliC*) were grown in SCFM2 with 2% mucin for 8 hours, then treated with tobramycin (300µg/mL) for 24 hours. Percent survival is plotted as mean +/- SD. **D)** Bacteria were grown in SCFM with 2% mucin (w/v) for 6 hours prior to imaging. **E)** Quantification of aggregates from **D**) using Imaris. **P<0.05,* as determined by **B)** unpaired student's t-test or **C,E)** one way ANOVA with Dunnett's post-hoc test. NS = not significant.

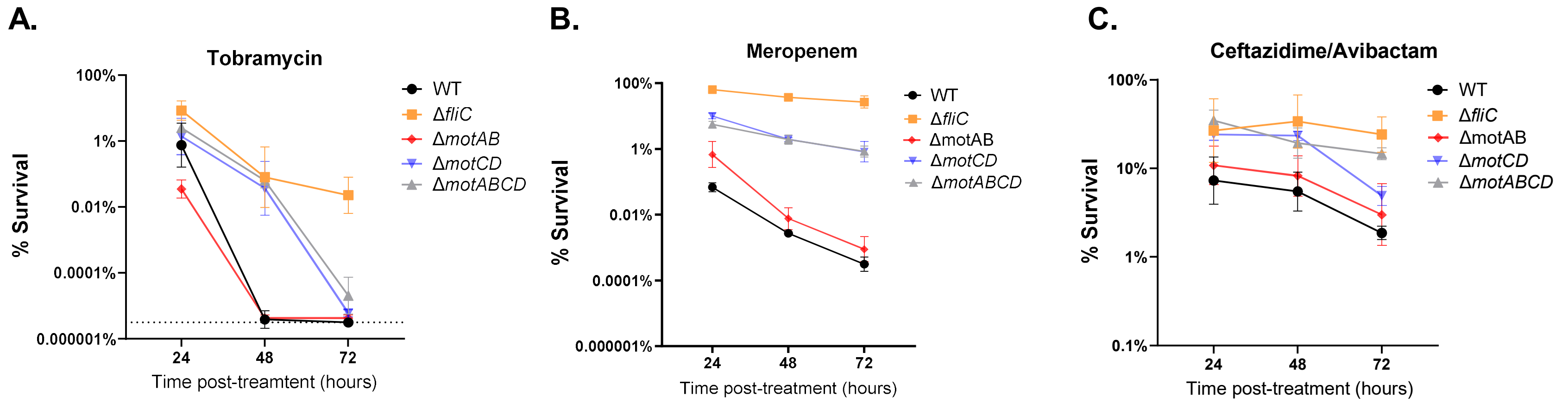

Figure S3. Loss of flagellar motility increases tolerance and persistence to multiple classes of antibiotics. Bacteria were grown for 8 hours in SCFM2 with 2% mucin (w/v) then treated with **A)** tobramycin (300μg/mL), **B)** meropenem (2000μg/mL), or **C)** ceftazidime/avibactam (1000/40 μg/mL) for up to 72 hours. Dashed line in (**A**) indicates limit of detection (100 CFU/mL).

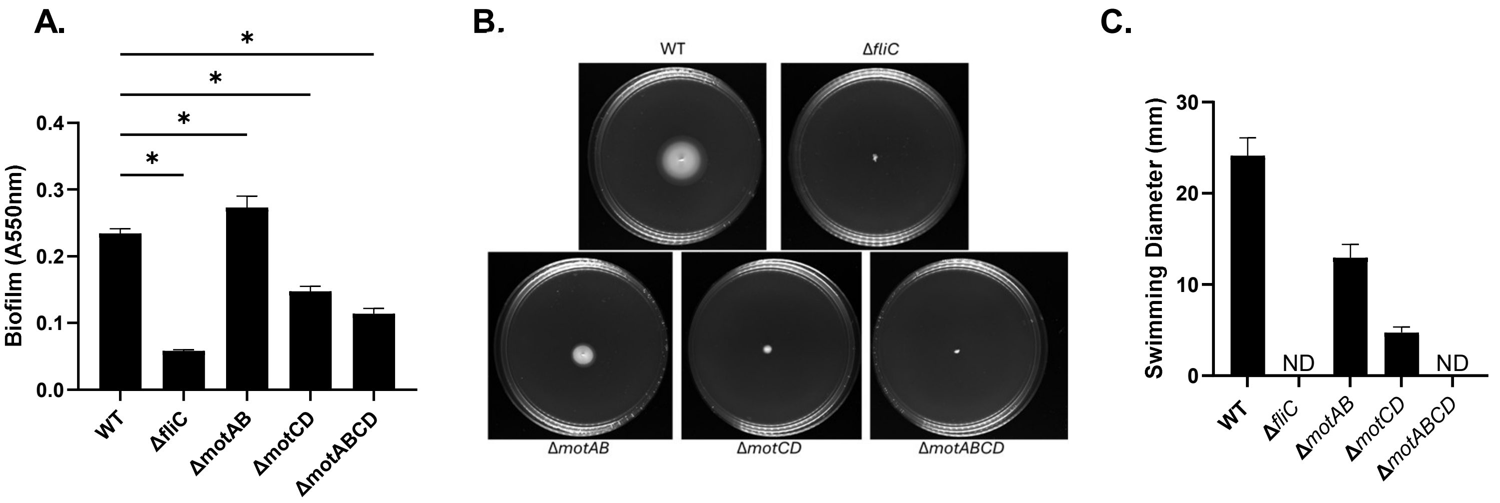

Figure S4. Biofilm formation and agar swimming motility of flagellar mutants. A) Bacteria were grown in SCFM2 with 2% mucin (w/v) for 24 hours before staining surface attached bacteria with 0.1% crystal violet. Absorbance was measured at 550nm. **B)** Bacteria were inoculated into LB with 0.3% agar and incubated at room temperature for ~30 hours before zone of motility was measured. Representative images of motility zones shown. **C)** Quantification of swimming motility from (**B**). ND = No zone of motility detected. **P<0.05* as determined by one way ANOVA with Dunnett's post-hoc test.

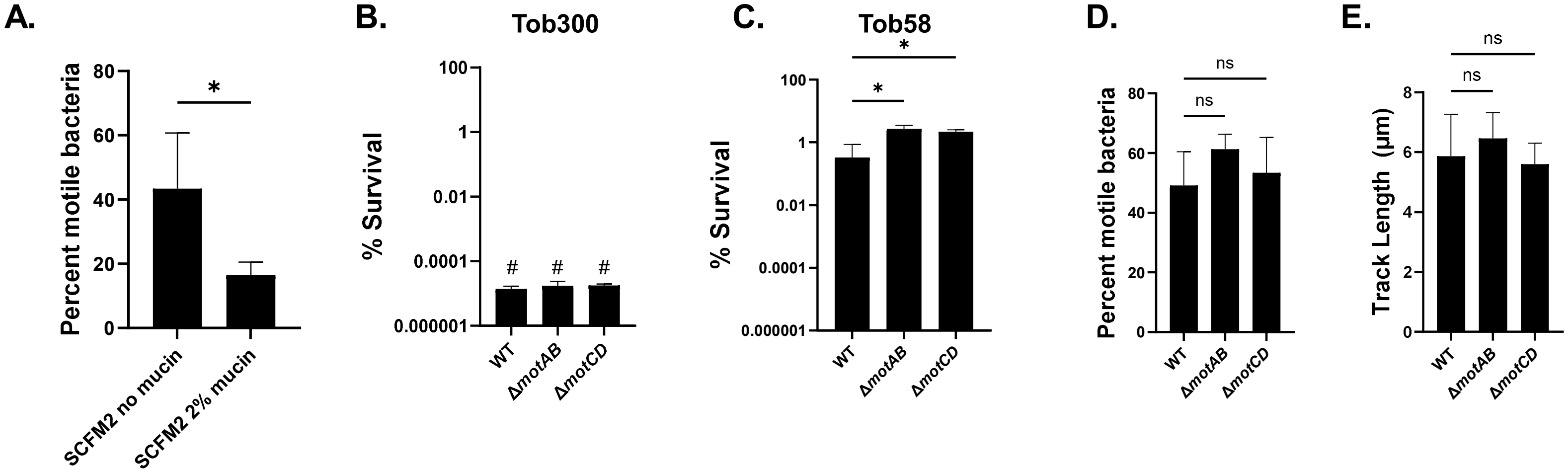

Figure S5. Mucin constrains motility. A) Single cell motility tracking for WT mPAO1 was conducted either in SCFM2 with 2% mucin or SCFM2 lacking mucin. The percent of motile bacteria is presented as mean +/- SEM. **B,C)** The indicated mPAO1 strains were grown in SCFM2 containing 0% mucin for 8 hours, then treated with **B)** 300 µg/mL tobramycin or **C)** 58µg/mL tobramycin for 24 hours. # indicates no recovered CFU. **D,E)** Single cell motility tracking was performed in SCFM2 without mucin and percent motile bacteria (**D**) or track length (**E**) was measured and plotted as mean +/- SD. *$P<0.05$ as determined by student's *t*-test (**A**), or one-way ANOVA with Dunnett's post-hoc test (**C-E**)**.** NS = not significant.

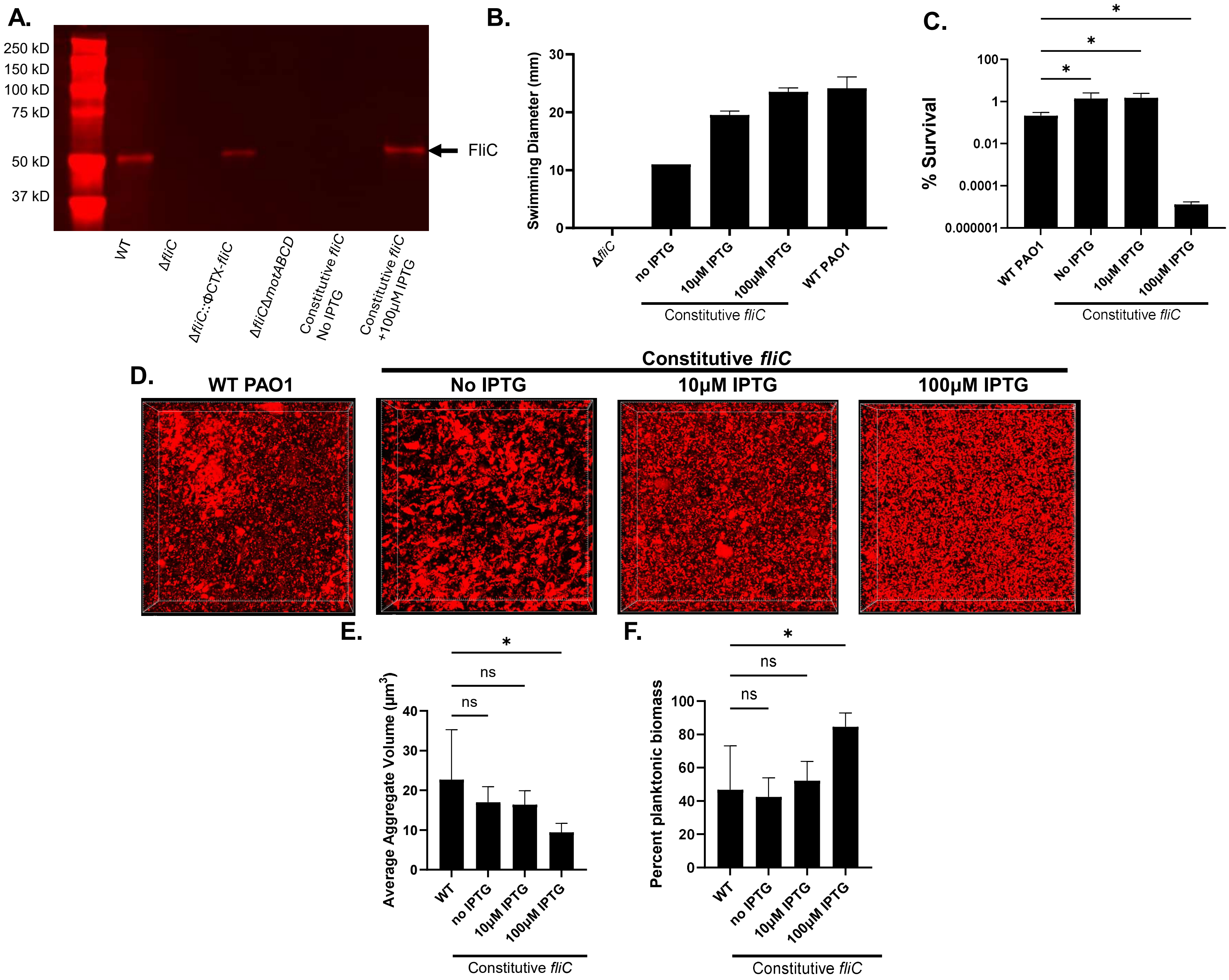

Figure S6. Inducible constitutive *fliC* expression increases tobramycin sensitivity. A) Western blot of surface flagella of indicated strains after growth in SCFM2 with 2% mucin (w/v). **B)** Soft agar motility assay of constitutive *fliC* strain at varying IPTG levels. **C)** Survival of WT or constitutive *fliC* strain (Δ*fliC*+pMMB-*fliC*) at varying IPTG levels to tobramycin (300μg/mL). **D)** Bacteria were grown in SCFM2 with 2% mucin for 6 hours prior to confocal imaging. **E)** Aggregate volumes from **D**, quantified using Imaris. **F)** Percent planktonic biomass from **D**. **P<0.05* as determined by one-way ANOVA with Dunnett's post-hoc test. NS = not significant.

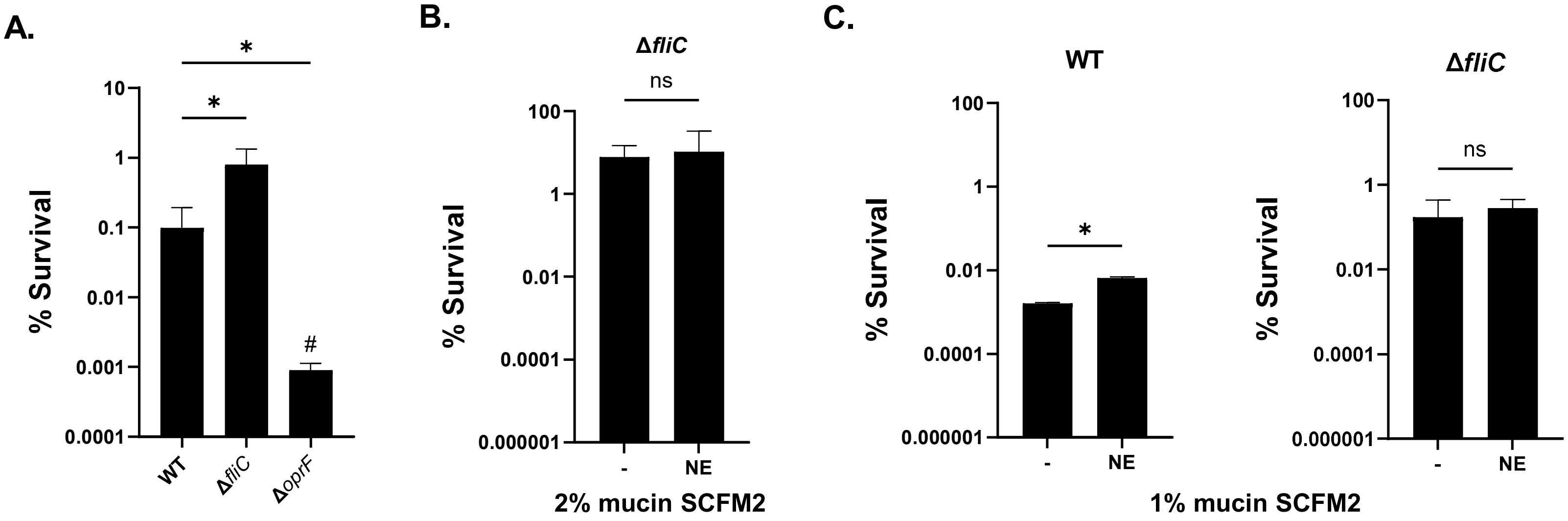

Figure S7. Promotion of tobramycin tolerance by neutrophil elastase is specific to flagellin. A) WT mPAO1, Δ*fliC,* or Δ*oprF* were grown in SCFM2 with 2% mucin (w/v) for 8 hours then treated with tobramycin (300μg/mL) for 24 hours. # indicates no recovered CFU. **B)** mPAO1 Δ*fliC* was grown for 8 hours in SCFM2 with 2% mucin with or without 150μg/mL neutrophil elastase (NE), then treated with tobramycin (300μg/mL) for 24 hours. **C)** WT or Δ*fliC m*PAO1 were grown for 8 hours in SCFM2 with 1% mucin with or without 150μg/mL neutrophil elastase (NE), then treated with tobramycin (300μg/mL) for 24 hours. **$P<0.05$* as determined by students *t*-test. NS = Not significant.

Table S1: MIC values of PAO1 strains used in this study in SCFM2 with varying mucin concentrations to tobramycin, meropenem, and ceftazidime. MIC were measured by broth microdilution using CLSI standards. MIC values (µg/mL) shown.

	Tobramycin			Meropenem			Ceftazidime		
	**0% mucin**	**1% mucin**	**2% mucin**	**0% mucin**	**1% mucin**	**2% mucin**	**0% mucin**	**1% mucin**	**2% mucin**
WT PAO1	2	2-4	8	4-32	8-32	8-32	4	4-8	2
Δ*fliC*	2	2-4	8	4-16	8-16	8-32	2-4	4-8	2-4
Δ*motAB*	2	2-4	8	4-16	8-32	8-32	2-4	2-4	2
Δ*motCD*	2	2-4	8	4-16	8-32	8-32	4-8	2-4	2
Δ*motABCD*	2	2-4	8	4-16	8-32	8-32	2-4	2-4	2
Constitutive *fliC*	2	2-4	8	8-16	8-32	8-32	2-8	2-4	1-2

Table S2. Strains and plasmids used in this study.

Strain/plasmid	Description	Source
Pseudomonas aeruginosa		
mPAO1	WT mPAO1	1
Δ*fliC*	In frame deletion of *fliC*	This study
Δ*flgE*	In frame deletion of *flgE*	This study
Δ*fliC*::ΦCTX-*fliC*	Native *fliC* complement via insertion of *fliC* and promoter into neutral ΦCTX site	This study
Δ*motAB*	In frame deletion of *motAB*	This study
Δ*motCD*	In frame deletion of *motCD*	This study
Δ*motABCD*	In frame deletion of *motABCD*	This study
WT mPAO1 *attTn7::dsRed-Express2*	WT mPAO1 expressing DsRed-express2 from Tn7 site	This study
Δ*fliC attTn7::dsRed-Express2*	Δ*fliC* strain expressing DsRed-express2 from Tn7 site	This study
Δ*fliC*::ΦCTX-*fliC attTn7::dsRed-Express2*	Native *fliC* complement strain expressing DsRed-Express2 from Tn7 site	
Δ*motAB attTn7::dsRed-Express2*	Δ*motAB* strain expressing DsRed-express2 from Tn7 site	This study
Δ*motCD attTn7::dsRed-Express2*	Δ*motCD* strain expressing DsRed-express2 from Tn7 site	This study
Δ*motABCD attTn7::dsRed-Express2*	Δ*motABCD* strain expressing DsRed-express2 from Tn7 site	This study
Δ*fliC*+pMMB-*fliC*	Constitutive *fliC* strain. Δ*fliC* strain carrying pMMB plasmid with *fliC* under TAC promoter.	This study
Δ*fliC*+pMMB-*fliC attTn7::dsRed-Express2*	Constitutive *fliC* strain expressing dsRed-Express2 at Tn7 site	This study
Δ*oprF*	In frame deletion of *oprF*	This study
Paer17	Motile CF clinical isolate	This study
Paer17 Δ*fliC*	In frame deletion of *fliC* in Paer17	This study
Paer35	Motile CF clinical isolate	This study
Paer35 Δ*fliC*	In frame deletion of *fliC* in Paer35	This study
Paer17 *attTn7::dsRed-Express2*	Paer17 expressing DsRed-express2 from Tn7 site	This study
Paer17 Δ*fliC attTn7::dsRed-Express2*	Paer17 Δ*fliC* expressing DsRed-express2 from Tn7 site	This study
Paer35 *attTn7::dsRed-Express2*	Paer35 expressing DsRed-express2 from Tn7 site	This study
Paer35 Δ*fliC attTn7::dsRed-Express2*	Paer35 Δ*fliC* expressing DsRed-express2 from Tn7 site	This study
**Plasmids**		
pDONR201	Gateway entry vector	Invitrogen
pEXG2	Suicide plasmid for in frame deletions	2
pFLP2	Vector containing flp recombinase for removing vector backbone of CTX and Tn7 plasmids	3
pTNS2	Helper plasmid for transformation of pUC18-mini-Tn7 vectors	4
pMMB67	IPTG inducible Expression vector	5
pMMB-*fliC*	pMMB67 with fliC ORF under TAC promoter	This study
pUC18T-mini-Tn7T-Gm-Pc-DsRed-Express2	Tn7 vector for insertion of DsRed-express2 fluorophore	6
pMini-CTX_GW	Gateway adapted CTX vector for chromosomal insertion	This study

Table S3. Primers used in this study.

Primer	Description	Sequence (5'-3')
fliC-up5'	Upstream homology arm for deletion of *fliC*	TACAAGAAAGCTGGGTGCCTTGAGAATGTCTTCGTTGGAAGAC
fliC-up3'		CCGGGCTTAGCGCAGCAGGCTCAGGTTGACTGTAAGGGCCATGGTGATTTC
fliC-down5'	Downstream homology arm for deletion of *fliC*	TACAAAAAAGCAGGCTGTGGACTGGGTGTTCTTCGGATTCTGC
fliC-down3'		GAAATCACCATGGCCCTTACAGTCAACCTGAGCCTGCTGCGCTAAGCCCGG
flgE-up5'	Upstream homology arm for deletion of *flgE*	TACAAAAAAGCAGGCTCAGAACGGCGAGTTCATCGCCCAACTG
flgE-up3'		CCGTCATCAGCGCAGGTTGATGATGGTCTGGTTGAAACTCATGGATAGCTCCTTGCC
flgE-down5'	Downstream homology arm for deletion of *flgE*	TACAAGAAAGCTGGGTCACCTTTTCCTCCGGCGGCACGGC
flgE-down3'		GGCAAGGAGCTATCCATGAGTTTCAACCAGACCATCATCAACCTGCGCTGATGACGG
motAB-up5'	Upstream homology arm for deletion of *motAB*	TACAAAAAAGCAGGCTGCGTTGCTGCCATTGCTCCAGTAG
motAB-up3'		CTTGATCTGCTCCAGCTTCAGGCTGCCCATGAGGACCGGACGTGCGAAATGAAC
motAB-down5'	Downstream homology arm for deletion of *motAB*	TACAAGAAAGCTGGGTGGCCTGGCGATGGACGAACTGCGC
motAB-down3'		GTTCATTTCGCACGTCCGGTCCTCATGGGCAGCCTGAAGCTGGAGCAGATCAAG
motCD-up5'	Upstream homology arm for deletion of *motCD*	TACAAAAAAGCAGGCTGCCTTCACCAAGGCCTTCGCCGAG
motCD-up3'		CGCAAACCATGGTTCGCGCGCTCATGGGACCAGGCTGAGCACATCCATCAGCGC
motCD-down5'	Downstream homology arm for deletion of *motCD*	TACAAGAAAGCTGGGTCAGCCCTTTCACCGCGAGGAACTC
motCD-down3'		GCGCTGATGGATGTGCTCAGCCTGGTCCCATGAGCGCGCGAACCATGGTTTGCG
Native *fliC*-5'	Amplification of *fliC* and promoter for native complement	TACAAAAAAGCAGGCTGTTGCACGGGAGGGCTAAAGAAAATCGCCG
Native *fliC*-3'		TACAAGAAAGCTGGGTTTCATTAGCGCAGCAGGCTCAGGACCGC
fliC gene-5'	For amplification of fliC gene for constitutive expression	AGGCTCGAGGAGGATATTCATGGCCCTTACAGTCAACACGAAC
fliC gene-3'		TACAAGAAAGCTGGGTTTCATTAGCGCAGCAGGCTCAGGACCGC
oprF-up5'	Upstream homology arm for deletion of *oprF*	TACAAAAAAGCAGGCTGCTTTCCGATGAAGAGCTGGTCGAGCG
oprF-up3'		CCTTAGAGGCTCAGCCGATTACTTGGCCCGTTAAATCCCCATCTTGATGGTCAG
oprF-down5'	Downstream homology arm for deletion of *oprF*	TACAAGAAAGCTGGGTCACGGGATAGACAGCGAGATAGTGAAC
oprF-down3'		CTGACCATCAAGATGGGGATTTAACGGGCCAAGTAATCGGCTGAGCCTCTAAGG
Paer17-*fliC*-up5'	Upstream homology arm for deletion of *fliC* in Paer17	TACAAAAAAGCAGGCTGAAGTCGACTGGGTGTTCTTCGGCATG
Paer17-*fliC*-up3'		CCTTGACCTCGCCGAAGCTTAGCGCATCTTGATGCCCTCCAAGGACGATATG
Paer17-*fliC*-down5'	Downstream homology arm for deletion of *fliC* in Paer17	TACAAGAAAGCTGGGTCTGGAACAGCTCGGGCTTGTTCAGTTC
Paer17-*fliC*-down3'		CATATCGTCCTTGGAGGGCATCAAGATGCGCTAAGCTTCGGCGAGGTCAAGG
Paer35-*fliC*-up5'	Upstream homology arm for deletion of *fliC* in Paer35	TACAAAAAAGCAGGCTCAGAACCGCTTCAACGAGTGCAAGAGC
Paer35-*fliC*-up3'		CGCGTGAGTGACCGTTCCCGGGCTTACATGGTGATTTCCTCCAAAGGACCTATTTC
Paer35-*fliC*-down5'	Downstream homology arm for deletion of *fliC* in Paer35	TACAAGAAAGCTGGGTGAACGCGCTGATCGCACTCTTGAACTG
Paer35-*fliC*-down3'		GAAATAGGTCCTTTGGAGGAAATCACCATGTAAGCCCGGGAACGGTCACTCACGCG

Supplemental references

1. Holloway BW. Genetic recombination in *Pseudomonas aeruginosa*. J Gen Microbiol. 1955 Dec;13(3):572–81.
2. Rietsch A, Vallet-Gely I, Dove SL, Mekalanos JJ. ExsE, a secreted regulator of type III secretion genes in *Pseudomonas aeruginosa*. *Proc Natl Acad Sci* USA. 2005 May 31;102(22):8006–11.
3. Hoang TT, Karkhoff-Schweizer RR, Kutchma AJ, Schweizer HP. A broad-host-range Flp-FRT recombination system for site-specific excision of chromosomally-located DNA sequences: application for isolation of unmarked *Pseudomonas aeruginosa* mutants. *Gene*. 1998 May 28;212(1):77–86.
4. Choi K-H, Schweizer HP. mini-Tn7 insertion in bacteria with single attTn7 sites: example *Pseudomonas aeruginosa*. *Nat Protoc*. 2006;1(1):153–61.
5. Fürste JP, Pansegrau W, Frank R, Blöcker H, Scholz P, Bagdasarian M, et al. Molecular cloning of the plasmid RP4 primase region in a multi-host-range tacP expression vector. *Gene*. 1986;48(1):119–31.
6. Miller WG, Leveau JH, Lindow SE. Improved gfp and inaZ broad-host-range promoter-probe vectors. *Mol Plant Microbe Interact*. 2000 Nov;13(11):1243–50.
